# A Machine Learning Model for the Prediction of Water Contact Angles on Solid Polymers

**DOI:** 10.1021/acs.jpcb.4c06608

**Published:** 2025-03-03

**Authors:** Jose Sena, Linus O. Johannissen, Jonny J. Blaker, Sam Hay

**Affiliations:** † Manchester Institute of Biotechnology and Department of Chemistry, The University of Manchester, Manchester M1 7DN, U.K.; ‡ Department of Materials and Henry Royce Institute, The University of Manchester, Manchester M13 9PL, U.K.; § Department of Biomaterials, Institute of Clinical Dentistry, University of Oslo, Oslo 0317, Norway

## Abstract

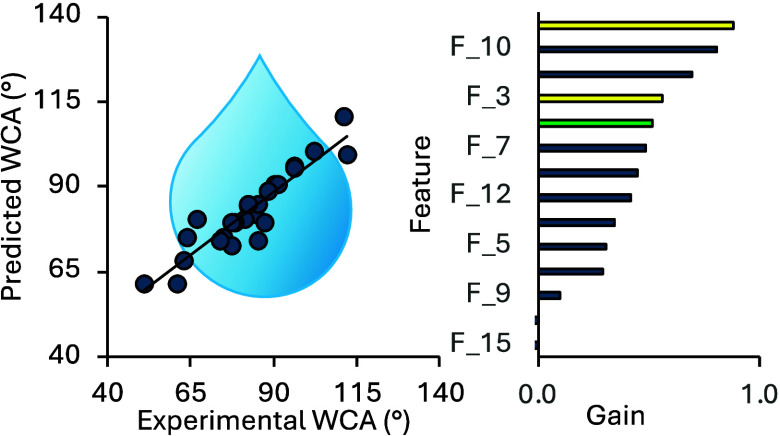

The interaction between water and solid surfaces is an active area of research, and the interaction can be generally defined as hydrophobic or hydrophilic depending on the level of wetting of the surface. This wetting level can be modified, among other methods, by applying coatings, which often modify the chemistry of the surface. With the increase in available computing power and computational algorithms, methods to develop new materials and coatings have shifted from being heavily experimental to including more theoretical approaches. In this work, we use a range of experimental and computational features to develop a supervised machine learning (ML) model using the XGBoost algorithm that can predict the water contact angle (WCA) on the surface of a range of solid polymers. The mean absolute error (MAE) of the predictions is below 5.0°. Models composed of only computational features were also explored with good results (MAE < 5.0°), suggesting that this approach could be used for the “bottom-up” computational design of new polymers and coatings with specific water contact angles.

## Introduction

Understanding the interaction of water with surfaces can be approached in diverse ways depending on the application. This interaction can be broadly categorized into two major categories, depending on the level of wetting of the surface: hydrophilic and hydrophobic. This property is derived from the interactions between water and the surface, and a key descriptor of this relationship is the angle formed between the surface and the water droplet; the water contact angle (WCA). Decades of research have shown how the interplay between the microstructure of the surface and its chemical composition affects surface properties.^1−5^ Thus, modifying the chemical composition and/or the surface microstructure allows a route to engineering materials with modified or controllable surface properties to tune materials properties such as waterproofing, anti-icing, self-cleaning, and antifouling.

With the increase in available computing power and computational algorithms, methods to develop new materials and coatings have shifted from being heavily experimental to including more theoretical approaches, with machine learning (ML) being a popular option. Lantada et al.^6^ created a database of microtextured surfaces to be used in conjunction with ML and machine vision algorithms to predict the effects of the microtextured on the material wettability. Yancheshme et al.^7^ explored the effect of water droplet shape on the water adhesion to a surface, for this, a random forest algorithm was used together with a plethora of physical features to develop an ML model that can predict the shape of a water droplet on a solid surface. Other applications of ML to predict chemical and materials properties include accelerated materials discovery,^8^ water droplet shape prediction,^7^ artificial intelligence-aided design,^6^ wettability of materials interfaces,^9^ and materials chemistry.^10^

Here, we describe a supervised machine learning model capable of predicting the water contact angle (WCA) of a diverse set of polymers using XGBoost^11^ (Table 1 and Figure S1). The model is trained on experimental data,^5,12−22^ atomic descriptors,^23^ and solvation-free energies computed using molecular dynamics (MD) simulations.^24^ Our results show good agreement between the predictions made by the model and the experimental values used for evaluation.

**Table 1:** Polymers Used in This Study^a^

polymer	category	WCA (deg)	in-house WCA (deg)^b^
polyether ether ketone (PEEK)	high-performance thermoplastic	68–94	68 ± 1
polyphenylene sulfide (PPS)	high-performance thermoplastic	75–87	75 ± 2
polysulfone (PSU)	high-performance thermoplastic	60–79	79 ± 6
polycarbonate (PC)	engineering thermoplastic	79–82	81 ± 5
polyethylene (PE)	standard thermoplastic	86–93	86 ± 2
polypropylene (PP)	standard thermoplastic	85–96	85 ± 2
polyethylene terephthalate (PET)	engineering thermoplastic	72–77	75 ± 1
poly(methyl methacrylate) (PMMA)	engineering thermoplastic	62–83	62 ± 2
poly(ethylene oxide) (PEO)	standard thermoplastic	63	–
acrylonitrile butadiene styrene (ABS)	copolymer	66–82	66 ± 1
fluorinated ethylene propylene (FEP)	copolymer	98–109	101 ± 1
polycaprolactam aramid 6 (Nylon-6)	engineering thermoplastic	63–69	69 ± 2
poly 1,3-butadiene (PBD)	standard thermoplastic	96	–
polychlorotrifluoroethylene (PCTFE)	halogen derivate of PE	80–99	80 ± 3
polyisobutylene (PIB)	standard thermoplastic	112	–
polystyrene (PS)	standard thermoplastic	72–86	86 ± 3
polytetrafluoroethylene (PTFE)	fluorinated derivate of PE	101–117	101 ± 3
polyvinyl acetate (PVA)	acetate derivate of PVC	60–61	–
polyvinyl alcohol (PVOH)	derivate of PVA	51	–
polyvinyl chloride (PVC)	standard thermoplastic	76–89	76 ± 2
polyvinyl fluoride (PVF)	fluorinated derivate of PVC	84–85	–
polyvinylidene fluoride (PVDF)	high-performance thermoplastic	73–88	73 ± 3
polyether sulfone (PES)	high-performance thermoplastic	73–90	73 ± 4
polyphenylene oxide (PPO)	high-performance thermoplastic	75–86	86 ± 2
styrene acrylonitrile (SA)	copolymer	74	–
polybutylene terephthalate (PBT)	engineering thermoplastic	85–88	85 ± 5

a Chemical structures are given in Figure S1 in the Supporting Information, WCAs are taken from the literature^5,12,13,18−22^ and in-house measurements.

b Polymers with unavailable experimental data are marked with “–”.

## Materials and Methods

### Sample Preparation

Pristine samples of 19 selected polymers (Table 1, Figure S1) were procured from Goodfellow Cambridge Limited, U.K. These were cleaned with isopropyl alcohol and a soft towel to prevent scratching the surface before measuring the static WCA of a 5 μL water droplet using the sessile drop technique with a Kruss DSA100 apparatus, and the surface roughness was measured using a Bruker Contour GTR-K1 Interferometer. Measured WCA and surface roughness values are given in Table S1 in the Supporting Information. In-house WCA measurements and those from literature sources are compared in Figure S2.

### MD Simulations

The molecular coordinates of each polymer were generated using the Avogadro software.^25^ The Antechamber package implemented in Ambertools 19^26^ was used to generate MD parameters, with the AM1-BCC method^27^ used to produce charges compatible with the general amber force field (GAFF) and solvated in water using the TIP3P model^28^ in a 2 nm octahedral box. Conversion from amber topology files to GROMACS topology files was performed using ParmED.^29^ GROMACS^30,31^ was used to minimize and equilibrate the structure before computing the solvation-free energy using the Bennet acceptance ratio (BAR) method, which is an algorithm that estimates the difference in free energy between two systems.^32^ For each molecule, the energy minimization and energy equilibration were carried out as follows: (i) steepest descent energy minimization with a maximum step size of 0.01 nm and a tolerance of 10.0 kJ mol^–1^ nm^–1^; (ii) 2 ns energy equilibration at 300 K using the sd integrator (Langevin mechanics). The equilibrated system was then used to produce the solvation-free energy using the thermodynamic integration procedure described in Shirts.^33^ We chose 30 equidistant steps for the coupling parameter λ, the sd integrator, and a simulation time of 2 ns at a temperature of 300 K and pressure of 1 bar. After this process is completed, the simulation results of each λ step are merged using the BAR algorithm to calculate the solvation-free energy.

### ML Model Evaluation Metrics

The ML model was evaluated according to (i) root mean square error (RMSE), (ii) mean absolute error (MAE), and (iii) coefficient of determination (*R*^2^) using the following equations:^34^
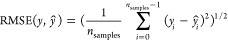
1

In eqs 1, 2, and 3, *ŷ* refers to the experimental values and *y* refers to the predicted values.
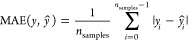
2

The mean absolute error is calculated as the sum of the differences between each predicted value and its corresponding experimental value. The quotient between this sum and the number of samples is the result.
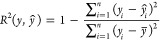
3

The coefficient of determination, usually denoted as *R*^2^, provides a measure of how good the prediction of unseen values would be. The maximum value of this metric is 1 and can also go to negative values as the model prediction can be arbitrarily poor.

## Results and Discussion

The 26 polymers examined in this study are composed of thermoplastic polymers, chemical relatives of these polymers, and three copolymers. These are given in Table 1. The thermoplastic polymers were selected due to their wide usage in the automotive and aerospace industries and the availability of samples for testing. The chemical relatives of the thermoplastic polymers were chosen for their chemical similarity. The distribution of the experimental contact angle for these materials is 66% hydrophilic (WCA < 90°) and 34% hydrophobic (WCA ≥ 90°).

The surface energy (γ) was selected as an ML feature due to its relationship with the WCA based on the theoretical work of Young, Wenzel, Cassie, and Baxter.^4,35,36^ The surface energy of the polymers and their static WCA values were obtained from various sources found in the literature^5,19−22^ and complemented with in-house measurements, which also included measurements of the surface roughness (Table S1). For polymers not available commercially as films or sheets, the WCA and surface roughness values were taken from the literature. Surface roughness was chosen as an additional experimental feature as this relates to the Wenzel, Cassie, and Baxter description of the WCA on real (nonideal) surfaces.

**Figure 1:**
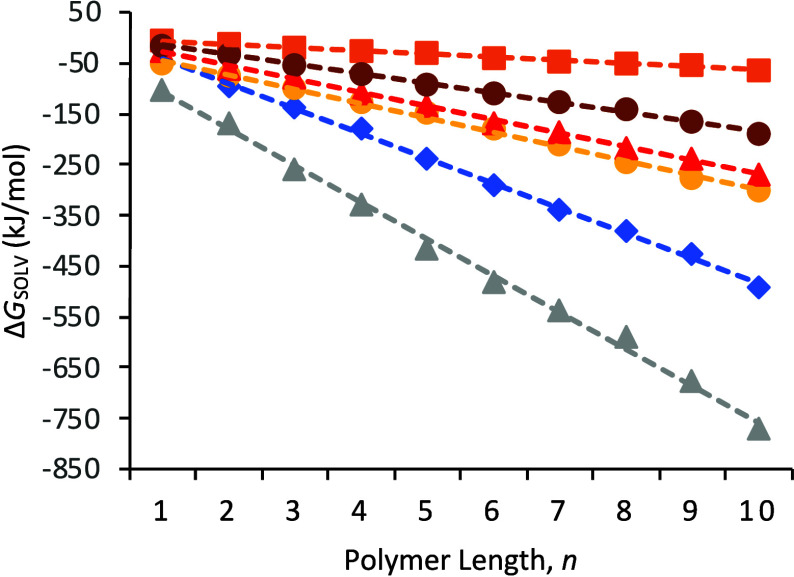
Solvation free energy, Δ*G*_solv_ computed at different polymer lengths for PPS (orange), PMMA (maroon), PET (red), PC (yellow), PEEK (blue), and PSU (gray). Linear fits are shown as dashed lines, and fitted values are given in Table S2.

The solvation-free energy (Δ*G*_solv_) was selected as another feature, as the relationship between Δ*G*_solv_ and hydrophobic surfaces has been explored previously,^37,38^ suggesting this is likely to be a useful feature for WCA prediction. These values were computed from MD simulations of each polymer within a TIP3 water box using a thermodynamic integration method.^32,33^ The polymer length selected for this process was determined from a series of Δ*G*_solv_ calculations performed on selected polymers ranging in length from 1 to 10 monomer units (Figure 1). These show a linear dependence of Δ*G*_solv_ on polymer length, so subsequent Δ*G*_solv_ calculations for each polymer in Table 1 were performed on trimers (length = 3). The computed solvation-free energies are given in Table S3.

Eleven additional molecular features were obtained using selected atomic descriptors computed using RDKit.^23,39^ These were chosen based on chemical intuition, and their relative importance is explored below. Initially, an ML model was developed using these combined features (listed in Table 2) using an artificial neural network implemented in TensorFlow.^40^ This model was composed of 3 hidden layers of 32, 16, and 8 nodes each, and one output, the WCA. The model allows tuning of a range of hyperparameters, including the activation function, number of nodes per layer, loss function, optimizer, and number of epochs. The activation function selected was ReLU,^41^ the loss function was a mean squared error and the ADAM optimizer.^42^ The ML training and evaluation data set were assessed using the *leave-one-out cross-validation* (LOOCV) method,^43^ where for each polymer an instance of the model was run where this polymer was absent from the training data set and was the only one present on the evaluation data set. While it is more computationally expensive than other validation methods, an attractive property of LOOCV is that it generally minimizes the chance of overfitting.^44−46^ We also used 10-fold cross-validation for some model testing. Optimization of each hyperparameter did not result in a model with acceptable performance, so a series of other supervised regression and learning algorithms were explored.

**Table 2:** List of Model 1 Features and Their Relative Importance Computed Using the Built-In Total Gain Algorithm in XGBoost

feature index^a^	feature	gain^b^
F_2	surface energy^c^	0.442
F_3	surface roughness^c^	0.048
F_4	solvation free energy^d^	0.035
F_5	sp^3^ hybridization^e^	0.008
F_6	sp^2^ hybridization^e^	0.022
F_7	fraction of sp^3^ C^e^	0.029
F_8	number of H-bond acceptors^e^	0.120
F_9	number of H-bond donors^e^	0.002
F_10	LogP^e^	0.257
F_11	number of non-H atoms^e^	0.008
F_12	average molecular weight^e^	0.018
F_13	molecular weight without H atoms^e^	0.000
F_14	number of H atoms^e^	0.011
F_15	number of valence electrons^e^	0.000

a F_1 is an identification value that is not used as an ML feature.

b Average Gain values of all models.

c Experimental values.

d Computed from MD simulations.

e Atomic descriptors were obtained using RDKit.

Partial least-squares regression, and the tree-based ML methods^44^ (Decision Tree, Random Forest, and XGBoost^11^) were used to build models that were trained on the polymers in Table 1. These models used the 14 features given in Table 2 to predict experimental WCA values, and the Python code for the XGBoost model is given in the Supporting Information. Training and evaluation data sets were created from data for the polymers in Table S4 and these data were normalized using the MinMaxScaler preprocessing module from the Sklearn library. The details of each model’s metrics are given in Table S5. From these models, the XGBoost method showed the best overall performance, so was selected as the preferred algorithm. The better overall performance of tree-base models over the neural network model may be attributed to the relatively small size of the data set and the tabular nature of the data.^44^

Next, the XGBoost hyperparameter values were optimized to improve the prediction performance (Table S6). A series of models were evaluated using the LOOCV method, where each predictor hyperparameter was systematically varied. A comparison with the default values from XGBoost is given in Table S7. In addition to improving the prediction accuracy, this process examines how the chosen hyperparameters perform on unseen data, providing opportunities to identify any cases of overfitting. We also performed k-fold cross-validation of this hyperparameter-optimized model, denoted “Model 1” to investigate model behavior (Figure S3). This shows the model smoothly improving in accuracy over ∼15 iterations, with no evidence for overfitting over 30 iterations, which is the same number used for the LOOCV models.

**Figure 2:**
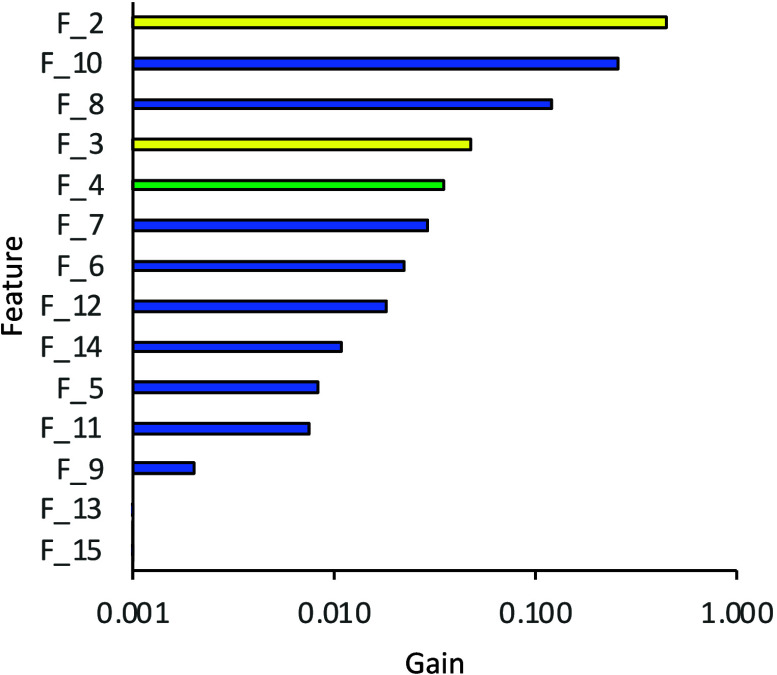
Total gain values for each feature from Table 2 in Model 1. RDKit features are shown in blue, surface energy and surface roughness in yellow, and the MD-computed Δ*G*_solv_ in green. A similar analysis performed using SHAP values^49^ is shown in Figures S4 and S5.

The relative importance of the features used for WCA prediction by Model 1 and their effect on the quality of the training was evaluated using the *total gain* function implemented in XGBoost.^47^ When this ML algorithm tries to split a leaf into two leaves, the gain is defined by the score on the original leaf, the score on the new leaf, and a regularization factor on the new leaf. The relative importance of each feature based on the total gain is shown in Figure 2. The most important features are found to be the surface energy (F_2) and partition coefficient (LogP, F_10). LogP is defined as the molecule distribution behavior in a biphasic system (commonly water/octanol), which helps predict absorption and other transport phenomena between liquid–liquid or liquid–solid layers.^48^ Two features: the average molecular weight without hydrogen atoms and the number of valence electrons, have the least effect on the training. The other experimental feature, surface roughness (F_3) is ranked fourth while the MD-computed solvation-free energy (Δ*G*_solv_, F_4) is ranked fifth, with both features having a similar gain value.

Comparison of the feature ranking between the XGBoost models with optimized and unoptimized hyperparameters shows some differences, which show how feature importance can be tuned through model optimization (Figure S4). In the unoptimized model, LogP is the highest-ranked feature, while the surface energy is ranked third, behind sp^2^ hybridization (F_6). The relative feature importance was also explored using Gini importance in the Decision Tree and Random Forrest models and Shapley Additive Explanation (SHAP)^49^ values. A comparison of feature ranking is given in Table 3. These analyses show that surface energy, logP, and surface roughness are consistently highly ranked, while the ranking of other features is more variable. To further explore feature importance, an XGBoost model was trained using only the top five features identified in Figure 2. This leads to a reduction in performance by 15% (Figure S6) but reduces the chance of overfitting in the model and demonstrates that this approach to WCA prediction is likely to be robust.

**Table 3:** Model Feature Ranking

	feature rank^a^				
model, method	1st	2nd	3rd	4th	5th
XGBoost Model 1, total gain	F_2	F_10	F_8	F_3	F_4
XGBoost Model 2, total gain	F_8	F_10	F_6	F_7	F_14
XGBoost,^b^ total gain	F_10	F_6	F_2	F_3	F_14
XGBoost,^b^ SHAP	F_2	F_10	F_4	F_3	F_6
Decision tree,^b^ Gini importance	F_2	F_10	F_11	F_3	F_11
Random Forrest,^b^ Gini importance	F_2	F_10	F_4	F_3	F_14

a Features are defined in Table 2 and Models 1 and 2 are presented in Figures 2 and 4.

b Model with default hyperparameter values and data taken from Figures S4 and S5.

**Figure 3:**
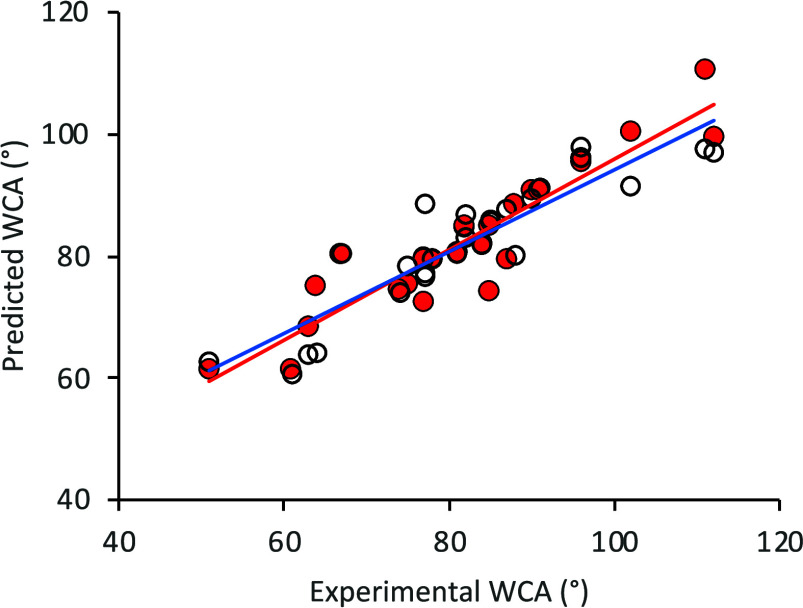
Output of the optimized XGBoost Model 1 (red) and Model 2 (blue), showing the predicted vs. experimental WCA for the 23 polymers and 3 copolymers listed in Table 1. The solid lines are a linear fit to the data with Model 1: *y* = 0.75*x* + 21.4 (*R*^2^ = 0.86) and Model 2: *y* = 0.67*x* + 27.1 (*R*^2^ = 0.82). Prediction metrics are given in Table 4.

The performance of Model 1 evaluated via the LOOCV method is also demonstrated in Figure 3, where the predicted WCA vs. experimental WCA values are compared. For comparison, linear regression of the value of each feature value vs. experimental WCA is shown in Figure S7. Surface energy has an *R*^2^ = 0.40 (cf. 0.86 for Model 1; Figure 3), while all other features have significantly smaller coefficients of determination. These data demonstrate the necessity of using e.g. an ML method with multiple features for WCA prediction, as single features do not correlate well with this property. The Model 1 performance was further evaluated using eq 1–3 and the evaluation metrics are detailed in Table 4. The average MAE shows a relatively small difference (<4.0°) between the experimental WCA value and the predicted value. However, the MAE metric does not provide the direction of the error, i.e., whether the model is under-predicting or over predicting the WCA, and inspection of Figure 3 shows values evenly distributed above and below the diagonal. The RMSE, like the MAE, is another metric that has the same units as the output, making it easy to interpret. Its main use is to manage the problem with small errors by virtue of the square root. Another benefit is this metric handling of outliers, which is smoother than the other metrics presented. The *R*^2^ coefficient of determination allows the percentage of the variation in the prediction to be traced to the variation in the inputs. The *R*^2^ value of 0.86 indicates that much of the variation in the predicted values can be traced back to the variation in the inputs.

**Table 4:** Prediction Metrics of Both Optimized XGBoost ML Models^a^

	RMSE (deg)	MAE (deg)	*R*^2^
model 1	5.7	3.7	0.86
model 2	6.5	4.1	0.82

a The values used to compute this metric are given in Tables S8 and S9, respectively.

Next, the ML model was retrained using only computational features to test the feasibility of this approach to predict the WCA of polymers where experimental data are unavailable, e.g., for polymers that are difficult to synthesize or for the optimization of new formulations. The surface energy and the surface roughness are the experimental data used as an input feature in the ML model, so “Model 2” was developed by retraining Model 1 with a data set that excludes these two features, using only the computed solvation free energy and RDKit atomic descriptors. While this approach removes two highly ranked features, we have demonstrated that optimization of hyperparameters can be used to alter feature ranking, suggesting that this approach may be successful. Model 2 was trained and tested using the same LOOCV method as Model 1 with reoptimization of hyperparameter values (Table S7).

**Figure 4:**
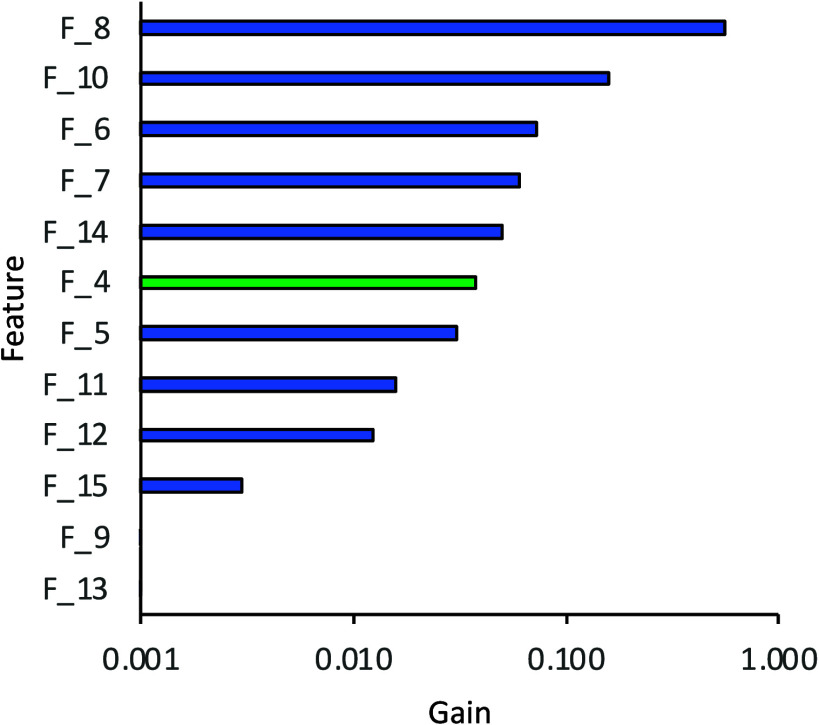
Total gain values for each feature in Model 2, determined using the total gain algorithm. RDKit features are shown in blue and the MD-computed Δ*G*_solv_ in green.

For Model 2, the relative feature importance, determined using the total gain, shifts in favor of the RDKit atomic descriptors (Figure 4 and Tables 3 and S10). The computed Δ*G*_solv_ values occupy the sixth position, while the number of hydrogen bond acceptors (F_8) is the highest-ranked feature for this model, with other important features largely overlapping with those found for Model 1 (excluding the missing experimental features). The prediction metrics of this model are compared to those of Model 1 in Table 4. The MAE is <5.0° and the *R*^2^ value is 0.82, which shows good performance, which is only modestly worse than that of Model 1. This shows the possibility of using this model when only computational data is available (either from simulations/modeling or molecular descriptors). The predicted vs. experimental WCA values for Model 2 are also shown in Figure 3. These data show a gradient of 0.67, which is comparable with the value of 0.75 for Model 1. The deviation of these gradients from unity shows the models systematically overpredicting lower WCA values and under-predicting higher WCA values. Adopting different scaling methods (other than min-max) did not rectify this, so future analysis, likely with a larger data set, is needed to investigate this behavior.

## Conclusions

In summary, a series of ML models were developed to predict the WCA on polymeric materials using a range of experimental and computational input parameters. XGBoost models were found to outperform other tree-based ML methods, as well as a neural network model. A range of computational features was found to be useful, including a selection of RDKit features, and the solvation free energy computed from MD simulations. The experimental surface energy and surface roughness were found to be useful training features, but as demonstrated in Model 2, not essential, and it is possible to train models with good performance (average error <5.0°) using only computational input features. To the best of our knowledge, there are no previous reports of methods capable of predicting the WCA on these materials. Further, this approach provides more evidence that ML methods have utility in linking molecular descriptors (features) to macroscopic properties (i.e., the WCA), and we suggest that this approach could be useful in the “bottom-up” computational prediction and design of new polymeric materials with predefined water-surface properties such as the WCA.

## Data Availability

All data supporting this study are provided in the Supporting Information accompanying this paper.

## Supporting Information Available

Additional experimental details and computational analysis, including the Python code used (PDF)

## Abbreviations

MAE: mean absolute error
ML: machine learning
RMSE: root-mean-square error; solvation free energy; surface energy, γ
WCA: water contact angle
