# Supplementary material for: A Machine Learning Model for the Prediction of Water Contact Angles on Solid Polymers
Source: J Phys Chem B. 2025 Mar 3;129(10):2739–45. doi: 10.1021/acs.jpcb.4c06608 (PMC11912489; doi:10.1021/acs.jpcb.4c06608)
Supplement: Supplementary file 1 — jp4c06608_si_001.pdf [file jp4c06608_si_001.pdf]

Supporting Information:

A Machine Learning Model for the Prediction of Water Contact Angles on Solid Polymers

Jose Sena,[a,b] Linus O. Johannissen,[a] Jonny J. Blaker,[b,c] Sam Hay[a*]

[a] *Manchester Institute of Biotechnology and Department of Chemistry, The University of Manchester, Manchester M1 7DN, United Kingdom.*

[b] *Department of Materials and Henry Royce Institute, The University of Manchester, Manchester M13 9PL, United Kingdom.*

[c] *Department of Biomaterials, Institute of Clinical Dentistry, University of Oslo, Oslo 0317, Norway*

Corresponding Authors:

*jonny.blaker@manchester.ac.uk

*sam.hay@manchester.ac.uk

Table S1. Static WCA and surface roughness values measured in the current study.

Polymer	Static WCA (°)	Surface roughness (Sa) (nm)
Polyether ether ketone (PEEK)	68 ± 1	52
Polyphenylene sulfide (PPS)	75 ± 2	318
Polysulfone (PSU)	79 ± 6	485
Polycarbonate (PC)	81 ± 5	66
Polyethylene (PE) (high density)	86 ± 2	243
Polypropylene (PP)	85 ± 2	89
Polyethylene terephthalate (PET)	75 ± 1	35
Polymethyl methacrylate (PMMA)	62 ± 2	33
Acrylonitrile butadiene styrene (ABS)	66 ± 1	96
Fluorinated ethylene propylene (FEP)	101 ± 1	140
Polycaprolactum aramid 6 (Nylon6)	69 ± 2	60
Polychlorotrifluoroethylene (PCTFE)	80 ± 3	74
Polystyrene (PS)	86 ± 3	33
Polytetrafluoroethylene (PTFE)	101 ± 3	416
Polyvinyl chloride (PVC) (unplasticized)	76 ± 2	43
Polyvinylidene fluoride (PVDF)	73 ± 3	62
Polyether sulfone (PES)	73 ± 4	71
Polyphenylene oxide (PPO)	86 ± 2	713
Polybutylene terephthalate (PBT)	85 ± 5	818

Table S2. Tabulated R^2 values and fit values for the data in **Figure 1.**

Polymer	R^2	Gradient (kJ/mol/n)
Polyether ether ketone (PEEK)	0.9986	-48.03
Polyphenylene Sulfide (PPS)	0.9979	-6.40
Polysulfone (PSU)	0.9963	-77.33
Polycarbonate (PC)	0.9971	-30.55
Polyethylene Terephthalate (PET)	0.9985	-27.031
Polymethyl methacrylate (PMMA)	0.9972	-18.39

Table S3. Solvation free energy (ΔG_{solv}) values computed for trimers of the polymers used in this study.

Polymer	ΔG_{solv} (kJ/mol)
Polyether ether ketone (PEEK)	-136.34
Polyphenylene Sulfide (PPS)	-149.17
Polysulfone (PSU)	-260.7
Polycarbonate (PC)	-99.45
Polyethylene (PE)	-29.12
Polypropylene (PP)	-56.43
Polyethylene Terephthalate (PET)	-299.64
Polymethyl methacrylate (PMMA)	-168.23
Polyethylene Oxide (PEO)	-85.04
Acrylonitrile Butadiene Styrene (ABS)	-329.55
Fluorinated Ethylene Propylene (FEP)	-156.09
Polycaprolactam Aramid 6 (Nylon 6)	-246.02
Poly 1,3-butadiene (PBD)	-58.76
Polychlorotrifluoroethylene (PCTFE)	-89.76
Polyisobutylene (PIB)	-67.91
Polystyrene (PS)	-146.97
Polytetrafluoroethylene (PTFE)	-69.51
Polyvinyl Acetate (PVA)	-143.93
Polyvinyl Alcohol (PVOH)	-102.39
Polyvinyl Chloride (PVC)	-68.05
Polyvinyl Fluoride (PVF)	-48.85
Polyvinylidene Fluoride (PVDF)	-60.77
Polyether Sulfone (PES)	-996.5
Polyphenylene Oxide (PPO)	-186.32
Styrene Acrylonitrile (SA)	-293.39
Polybutylene Terephthalate (PBT)	-393.97

Table S4. Complete dataset used for the training and test of the model using the LOOCV method. Features are described in **Table 2**.

F_1*	F_2	F_3	F_4	F_5	F_6	F_7	F_8	F_9	F_10	F_11	F_12	F_13	F_14	F_15	WCA
0	33	52	136	6	33	0.29	9	0	6.06	72	955	901	54	360	82
1	40	318	149	3	18	0.08	3	3	3	21	331	313	18	108	84
2	44	485	260	12	78	0.36	12	3	6.82	90	1256	1189	67	456	67
3	45	66	99.	9	48	0.56	9	3	4.28	57	769	721	48	294	81
4	32	243	29	6	0	1	0	0	3.62	6	86	72	14	38	90
5	29	89	56	10	0	1	0	0	4.74	10	142	120	22	62	91
6	44	35	299	6	36	0.6	12	0	3.62	42	583	553	30	222	75
7	40	33	168	12	9	0.8	6	0	1.84	21	306	276	30	126	77
8	42	150	85	4	0	1	6	6	0	12	186	168	18	78	63
9	35	96	329	1	12	0.07	3	0	4.75	45	586	547	39	222	78
10	20	140	156	17	0	1	0	0	5.05	51	864	864	0	312	102
11	43	60	246	6	2	0.83	6	3	1.73	24	346	307	39	144	64
12	32	150	58	2	2	0.5	0	0	2.61	12	168	144	24	72	96
13	31	74	89	6	0	1	0	0	2.81	18	355	349	6	114	96
14	32	259	67	4	0	1	0	0	2.69	12	174	144	30	78	112
15	38	33	146	0	8	0	0	0	3.36	24	312	288	24	120	77
16	21	416	69	6	0	1	0	0	2.55	18	306	300	6	114	111
17	39	7	143	3	3	0.75	6	0	1.6	18	264	240	24	108	61
18	37	11	102	3	0	1	3	3	1.03	9	138	120	18	60	51
19	40	43	68	3	0	1	0	0	2.27	9	194	179	15	60	85
20	28	40	48	3	0	1	0	0	2	9	144	129	15	60	85
21	27	62	60	4	0	1	0	0	2.3	12	198	186	12	78	82
22	46	71	996	2	30	0.06	18	3	6.1	96	1400	1345	55	486	87
23	47	713	186	0	7	0	3	3	2.42	21	282	264	18	108	77
24	40	8	293	4	8	0.36	3	3	4.25	36	484	439	45	192	74
25	32	818	393	5	11	0.33	12	3	2.46	48	667	625	42	258	88

*F_1 the Polymer index given in **Table S3**. This feature was not used in the training or validation of Model 1 nor Model 2.

Table S5. Metrics comparing different regression and ML methods. The presented metrics are for unoptimized models, using default parameters for each model. The scoring convention is that lower RMSE and MAE values are better, while R^2 that approach unity are best.

Model name	RMSE	MAE	R^2
PLS[a]	22.8	21.3	-0.47
Decision tree	14.0	12.0	0.44
Random forest	11.9	9.3	0.60
Neural network	15.6	12.9	0.33
XGBoost	13.1	10.5	0.68

[a] Partial least squares regression.

Table S6. Hyperparameter values used to optimize each XGBoost model prediction.

Model 1

Polymer	Index	Number of estimators	Maximum tree depth	Learning rate
PEEK	0	100	1	0.2
PPS	1	100	1	0.2
PSU	2	10	2	0.1
PC	3	10	1	0.6
PE	4	15	1	0.1
PP	5	15	1	0.1
PET	6	100	6	0.3
PMMA	7	10	1	0.1
PEO	8	10	2	0.4
ABS	9	10	1	0.1
FEP	10	10	1	0.7
NYLON6	11	60	1	0.7
PBD	12	60	1	0.7
PCTFE	13	50	2	0.1
PIB	14	10	3	0.7
PS	15	10	4	0.1
PTFE	16	100	6	0.4
PVA	17	10	3	0.5
PVOH	18	100	5	0.4
PVC	19	100	1	0.7
PVF	20	20	2	0.3
PVDF	21	10	6	0.2
PES	22	10	2	0.1
PPO	23	10	1	0.1
SA	24	100	2	0.3
PBT	25	100	1	0.1

Model 2

Polymer	Index	Number of estimators	Maximum tree depth	Learning rate
PEEK	0	50	3	0.1
PPS	1	10	2	0.2
PSU	2	10	1	0.5
PC	3	10	1	0.2
PE	4	50	1	0.2
PP	5	100	3	0.2
PET	6	50	2	0.2
PMMA	7	10	3	0.1
PEO	8	50	1	0.2
ABS	9	100	2	0.1
FEP	10	10	1	0.5
NYLON6	11	100	5	0.1
PBD	12	100	2	0.1
PCTFE	13	50	1	0.1
PIB	14	100	4	0.1
PS	15	10	1	0.1

PTFE	16	50	2	0.2
PVA	17	100	2	0.2
PVOH	18	100	4	0.1
PVC	19	50	1	0.2
PVF	20	50	1	0.3
PVDF	21	10	1	0.1
PES	22	100	5	0.3
PPO	23	100	1	0.1
SA	24	100	4	0.2
PBT	25	100	1	0.3

The optimization was carried out with a python script that search a wide values space of one hyperparameter at the time using the root mean square error as the function to be minimized. The output of this scripts is used to select the best fit for one of the hyperparameters at the time, and by repeating this operation with the other two hyperparameters it gives a good direction for the optimization.

Table S7. Comparison between the hyperparameters of Model 1 and Model 2 with the default values used by XGBoost.

	Number of estimators (avg)	Maximum tree depth (avg)	Learning rate (avg)
Model 1	44	2	0.3
Model 2	60	2	0.2
Default values	100	6	0.3

Table S8. Output of Model 1. The predicted values were computed using experimental and computational features (**Table 2**), these values also presented in **Figure 3** (red).

Polymer	Index (F_1)	Experimental values	Predicted values	Difference (Exp. – Pred.)
PEEK	0	82	84.53	2.53
PPS	1	84	81.94	-2.06
PSU	2	67	80.36	13.36
PC	3	81	80.61	-0.39
PE	4	90	90.79	0.79
PP	5	91	90.75	-0.25
PET	6	75	75.23	0.23
PMMA	7	77	72.50	-4.50
PEO	8	63	68.23	5.23
ABS	9	78	79.44	1.44
FEP	10	102	100.36	-1.64
NYLON6	11	64	75.20	11.20
PBD	12	96	96.07	0.07
PCTFE	13	96	95.40	-0.60
PIB	14	112	99.62	-12.38
PS	15	77	79.71	2.71
PTFE	16	111	110.49	-0.52
PVA	17	61	61.41	0.41
PVOH	18	51	61.43	10.43
PVC	19	85	74.13	-10.87
PVF	20	85	84.88	-0.13
PVDF	21	82	85.08	3.08
PES	22	87	79.35	-7.65
PPO	23	77	79.51	2.51
SA	24	74	74.33	0.33
PBT	25	88	88.57	0.57

* F_1 is Polymer index, a number to identify the polymers. This feature was not used in the training or validation of Model 1 nor Model 2.

Table S9. Output of Model 2. The predicted values were computed using computational features only, these values also presented in **Figure 3** (blue).

Polymer	Index (F_1)	Experimental values	Predicted values	Difference (Exp. – Pred.)
PEEK	0	82	83.00	1.00
PPS	1	84	81.78	-2.22
PSU	2	67	80.50	13.50
PC	3	81	80.47	-0.53
PE	4	90	89.45	-0.55
PP	5	91	91.10	0.10
PET	6	75	78.27	3.27
PMMA	7	77	76.69	-0.31
PEO	8	63	63.90	0.90
ABS	9	78	79.65	1.65
FEP	10	102	91.37	-10.63
NYLON6	11	64	64.06	0.06
PBD	12	96	96.23	0.23
PCTFE	13	96	97.87	1.87
PIB	14	112	97.01	-14.99
PS	15	77	88.52	11.52
PTFE	16	111	97.47	-13.53
PVA	17	61	60.59	-0.41
PVOH	18	51	62.82	11.82
PVC	19	85	85.93	0.93
PVF	20	85	85.62	0.62
PVDF	21	82	86.91	4.91
PES	22	87	87.68	0.68
PPO	23	77	77.37	0.37
SA	24	74	73.91	-0.09
PBT	25	88	80.13	-7.87

Table S10. List of computational input features (**Model 2**) and their relative importance computed using the built-in total gain algorithm on XGBoost.

Feature index [a]	Feature	Gain [b]
F_4	Solvation free energy [c]	0.038
F_5	sp^3 hybridization [d]	0.030
F_6	sp^2 hybridization [d]	0.072
F_7	Fraction of SP3 C [d]	0.060
F_8	Number of H-bond acceptors [d]	0.559
F_9	Number of H-bond donors [d]	0.000
F_10	LogP [d]	0.160
F_11	Number of non-H atoms [d]	0.016
F_12	Average molecular weight [d]	0.012
F_13	Molecular weight w/o H atoms [d]	0.000
F_14	Number of H atoms [d]	0.050
F_15	Number of valence e^- [d]	0.003

[a] F_1 is an identification value that is not used as a ML feature. [b] Average Gain values of all computational models. [c] Computed from MD simulations. [d] Atomic descriptors obtained using RDKit. These values are tabulated in **Table S2**.

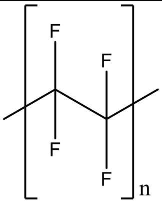

Polytetrafluoroethylene
(PTFE)

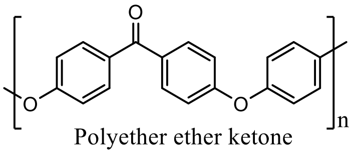

Polyether ether ketone
(PEEK)

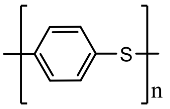

Polyphenylene sulfide
(PPS)

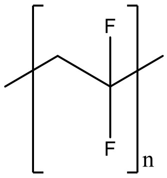

Polyvinylidene fluoride
(PVDF)

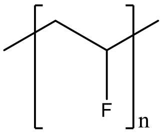

Polyvinyl fluoride
(PVF)

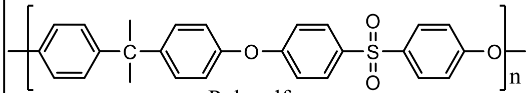

Polysulfone
(PSU)

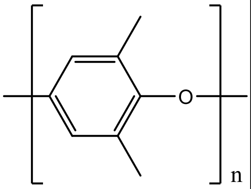

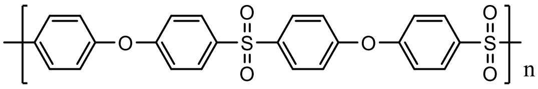

Polyethersulfone
(PES)

Poly(p-phenylene oxide)
(PPO)

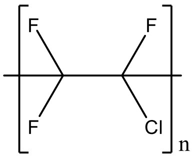

Polychlorotrifluoroethylene
(PCTFE)

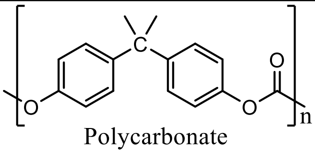

Polycarbonate
(PC)

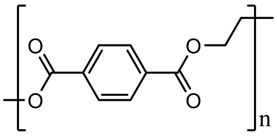

Polyethylene terephthalate
(PET)

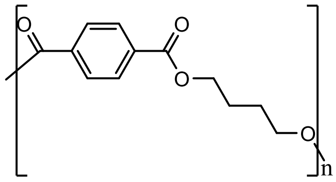

Polybutylene terephthalate
(PBT)

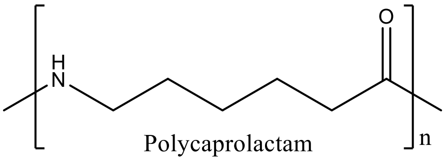

Polycaprolactam
(Nylon 6)

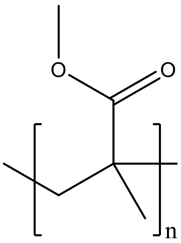

Poly(methyl methacrylate)
(PMMA)

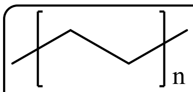

Polyethylene
(PE)

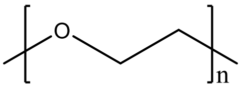

Polyethylene oxide
(PEO)

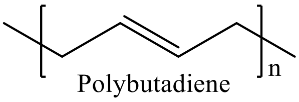

Polybutadiene
(PBD)

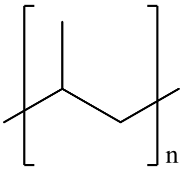

Polypropylene
(PP)

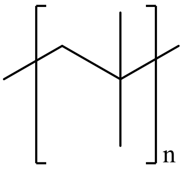

Polyisobutene
(PIB)

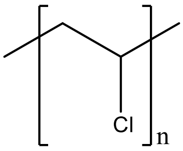

Polyvinyl chloride
(PVC)

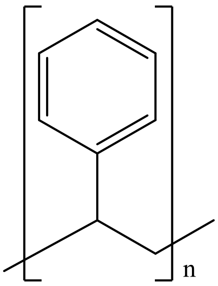

Polystyrene
(PS)

Continued next page.

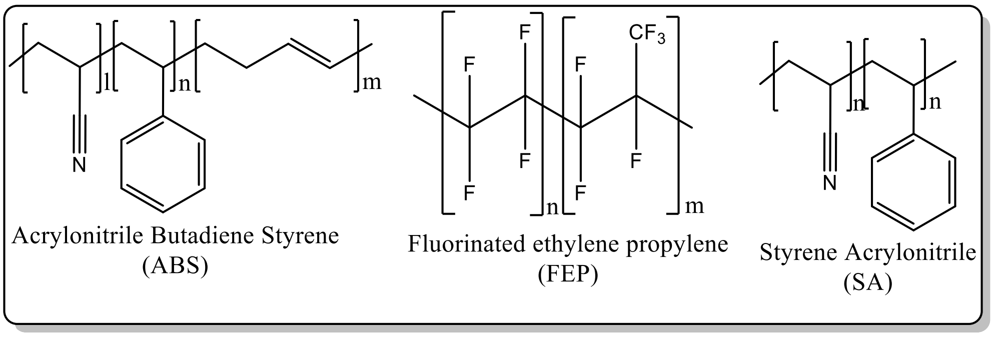

Figure S1. The molecular structure of polymers used in this study.

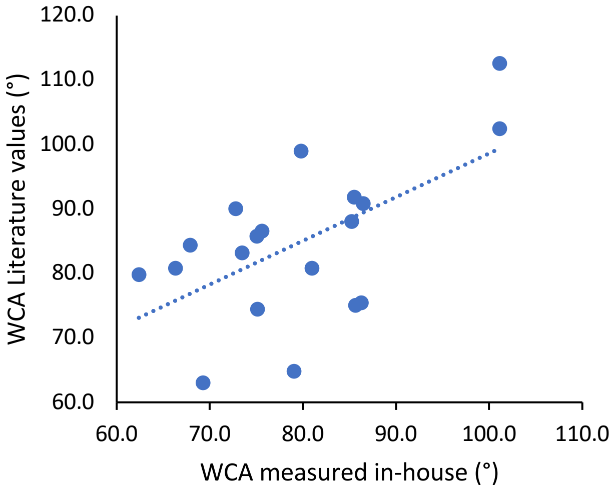

Figure S2. Comparison of WCA values obtained in house via the sessile drop technique with values obtained from the literature [1-8]. The in-house measurements are an average of five (5) different measurements, while the literature values are the average of all the values obtained from literature for each polymer.

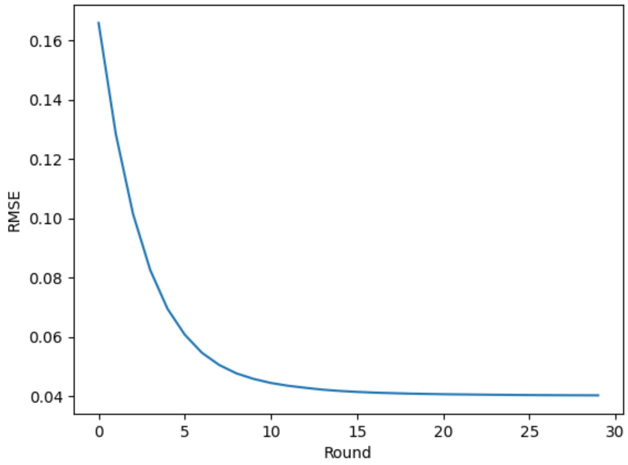

Figure S3. k-fold cross validation of the XGBoost model. The number of iterations (rounds) was 30, with 10-fold cross-validations. The learning rate was 0.3, which is the average used for the LOOCV-version of model 1. The metric optimized was RMSE, and the smooth reduction in RMSE with iteration shows that the model is well behaved and does not show evidence of overfitting.

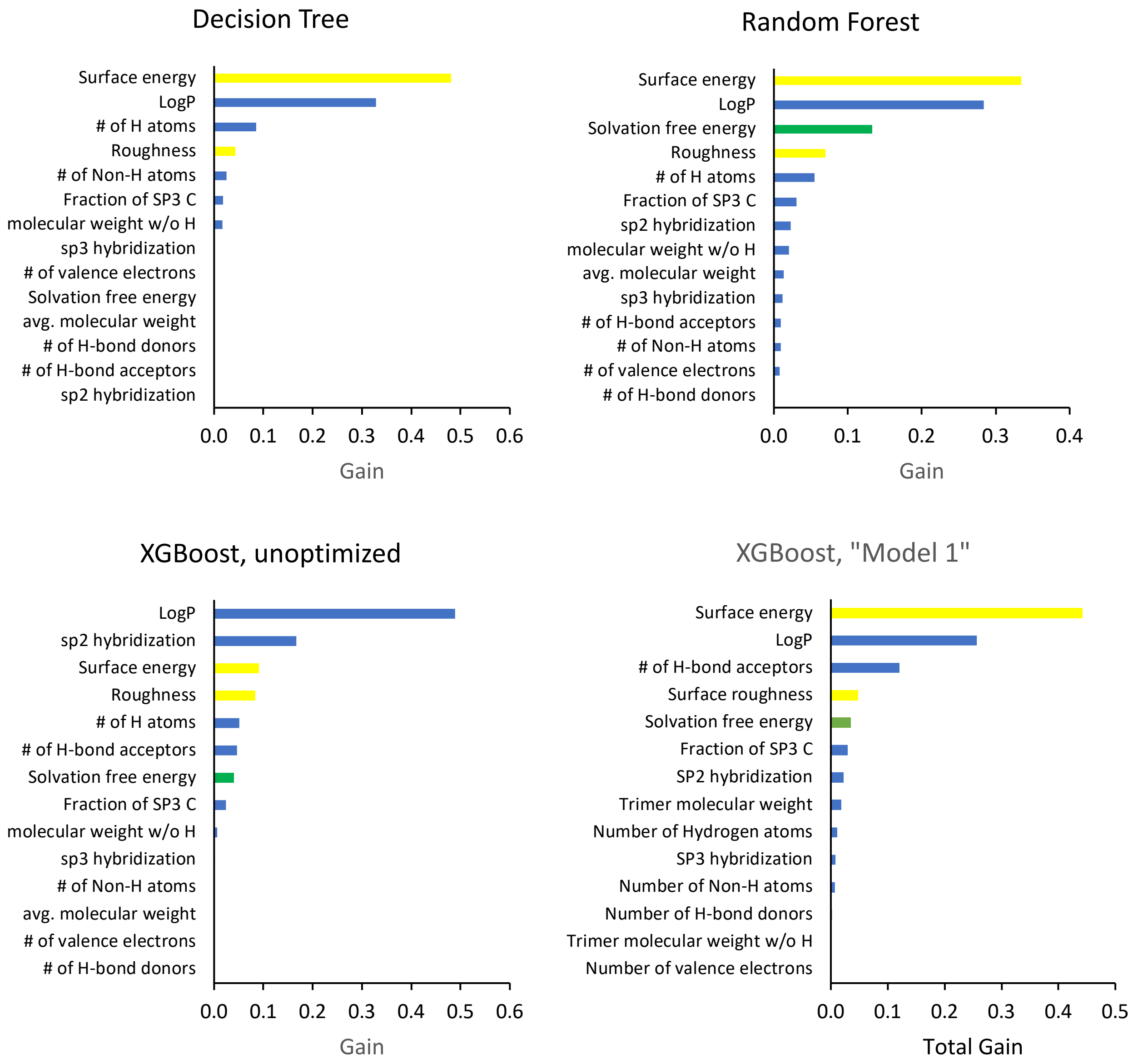

Figure S4. Feature importance for three tree-based ML models trained on the data in **Table S2** using the LOOCV method. The values plotted are from unoptimized models, and compared to the optimized XGBoost Model 1, which is reproduced from **Figure 2** in the main text. The Decision Tree and Random Forest models use the Gini importance to compute the relative feature importances, while XGBoost uses the Total Gain method. The same color coding is used as in **Figure 2**, with RDKit features shown in blue, surface energy and surface roughness in yellow and the MD-computed ΔG_{solv} in green.

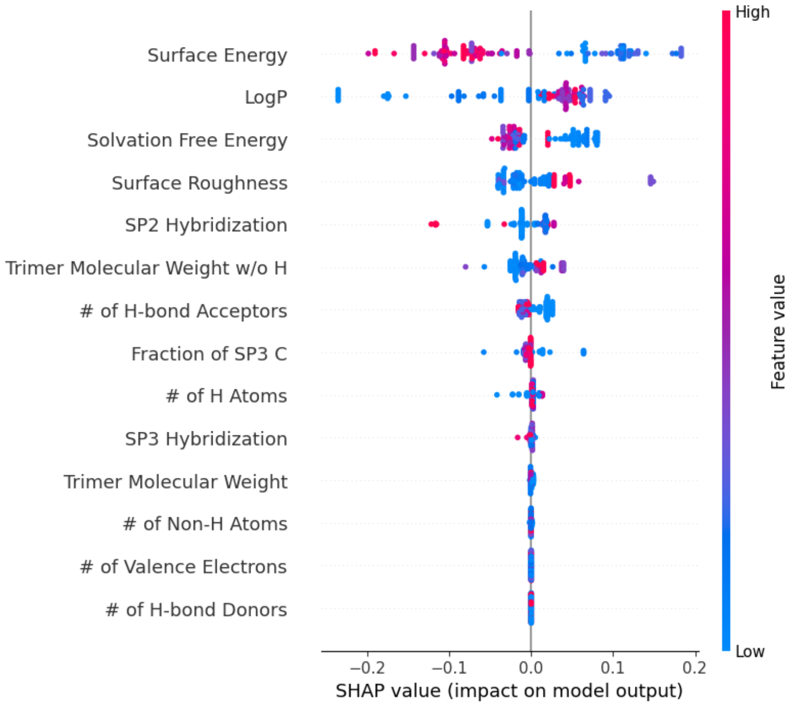

Figure S5. Feature importance of the XGBoost model in **Figure S4** investigated using SHAP values.[9] Most of the highest ranked features are similarly ranked in the XGBoost model, including: surface energy (SHAP: 1, Gain: 3), LogP (SHAP: 2, Gain: 1), solvation free energy (SHAP: 3, Gain: 6), Surface roughness (SHAP: 4, Gain: 4) and sp^2 hybridization (SHAP: 5, Gain: 2). These data also show that surface energy has large negative contribution to larger WCA values and a large positive contribution to smaller WCA values. Similarly, solvation free energy has a negative contribution to larger WCA values and a positive contribution to smaller WCA values. Conversely, positive LogP and surface roughness values have a positive contribution to larger WCA values, whereas smaller WCA values have more variable LogP and roughness values.

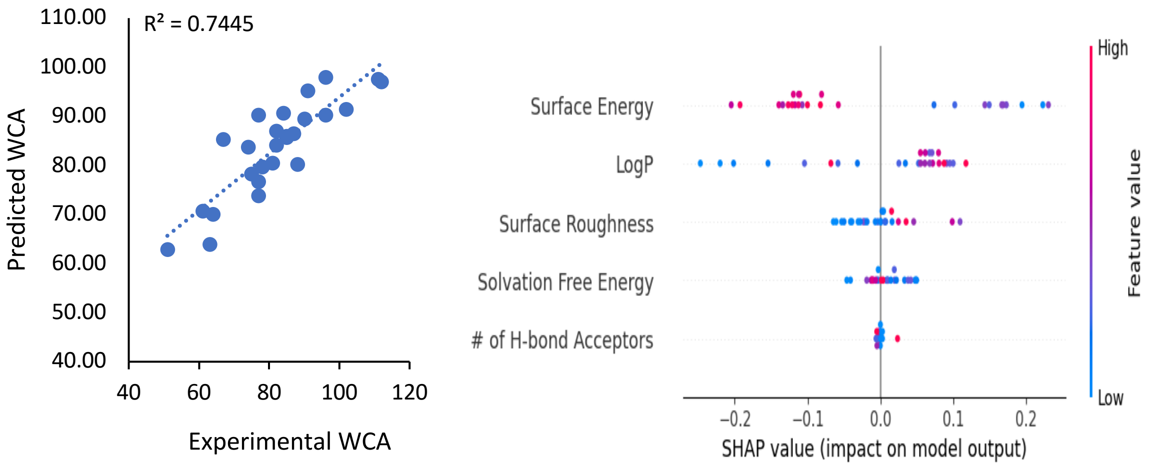

Figure S6. Output of the ML model 1 trained using only the top 5 features from the total gain results in **Figure 2**. For comparison, when the model is trained with all the features, it has an improvement in performance of 15%.

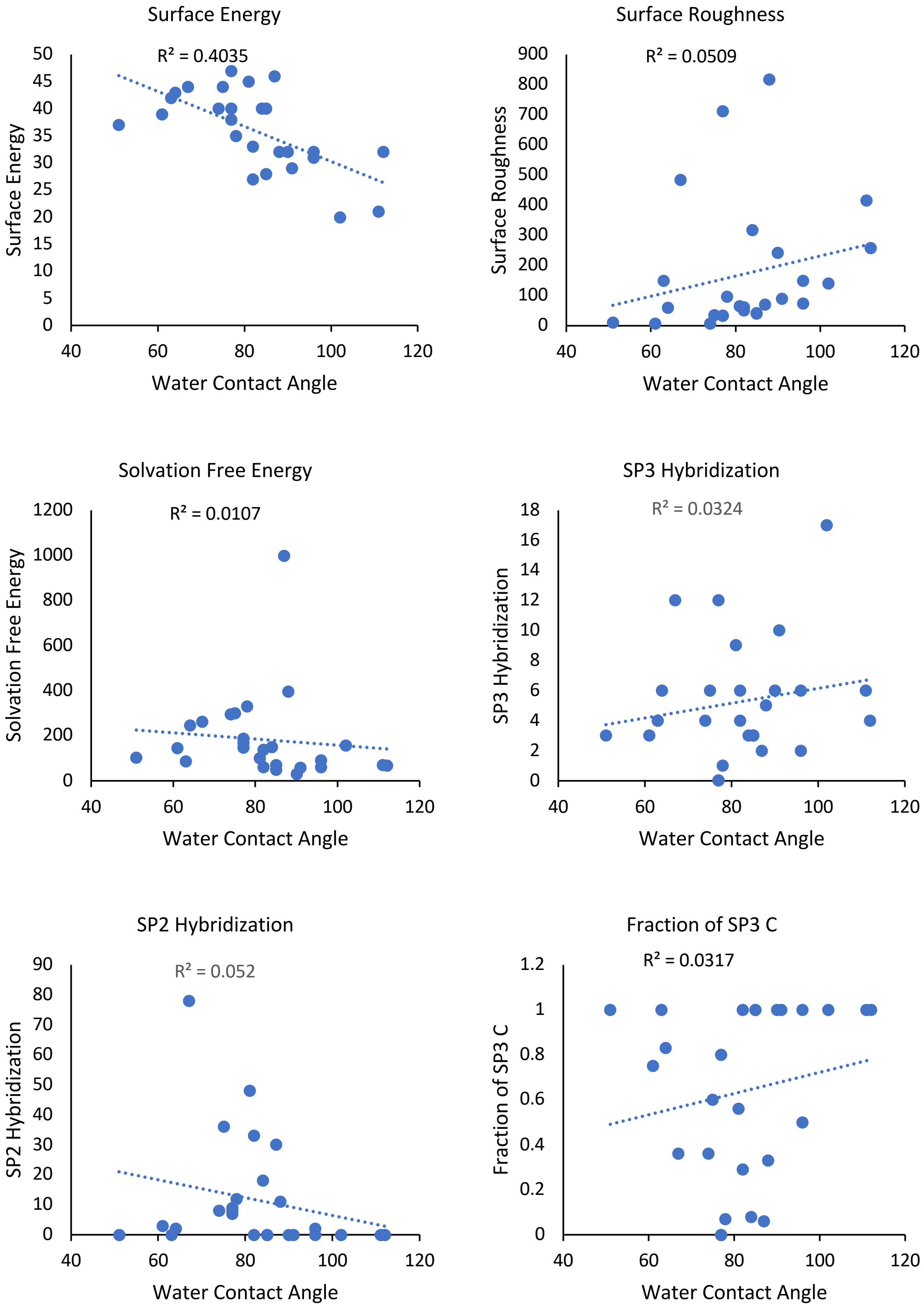

Continued next page.

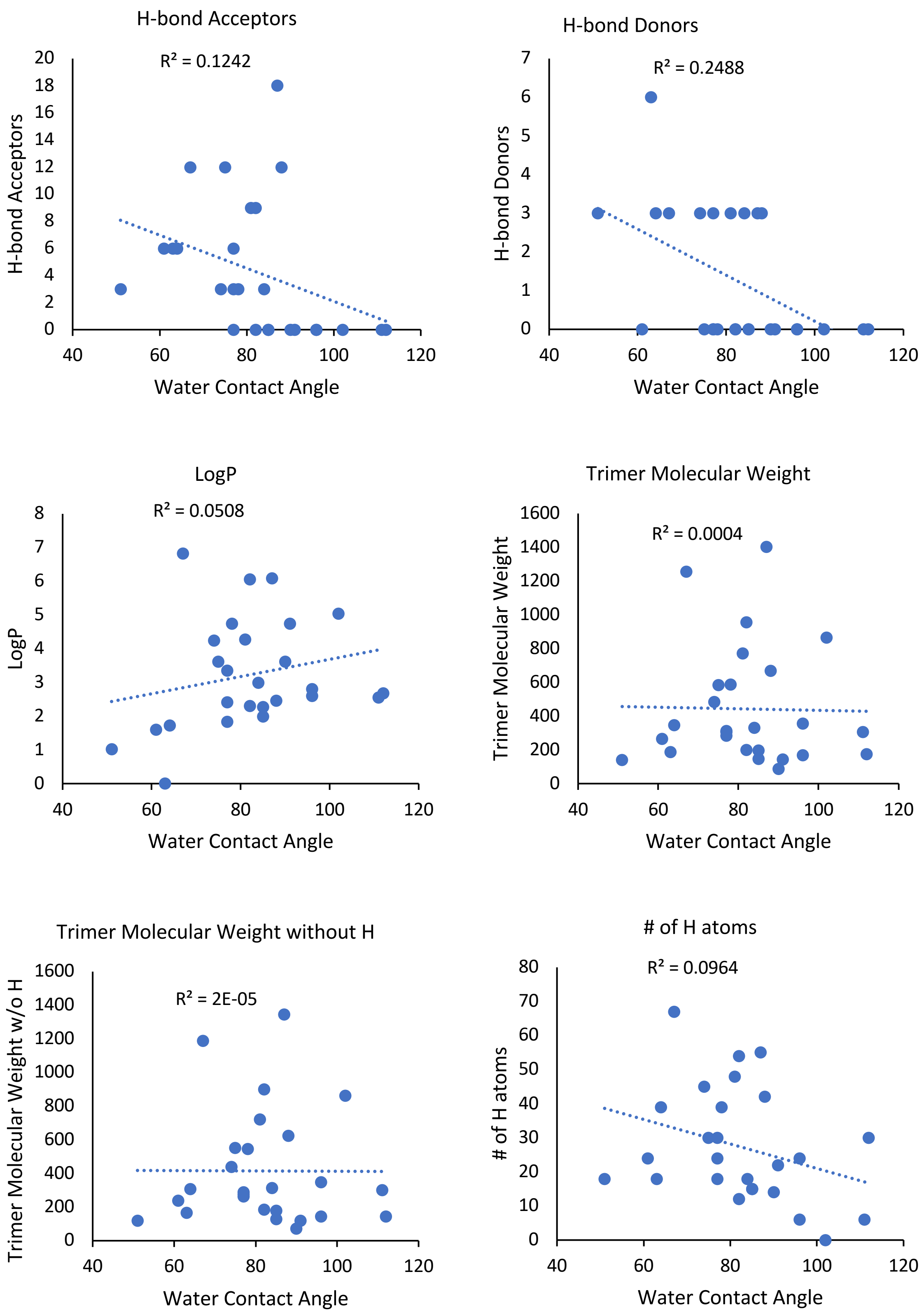

Continued next page.

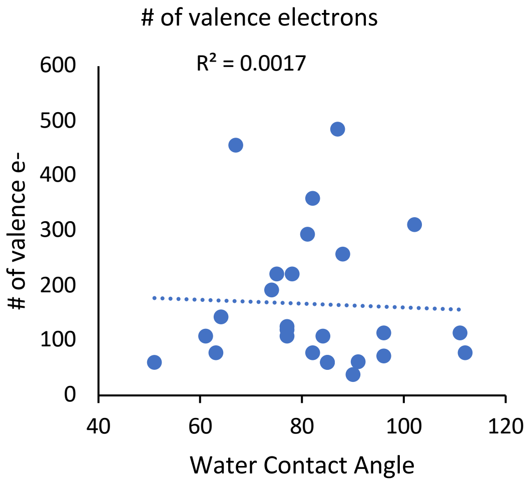

Figure S7. Linear correlation analysis of each feature vs. the experimental WCA. Data are taken from **Table S4**.

Supplementary Material 2. Surface roughness measurements:

Polyether ether ketone (PEEK), Sa = 52nm

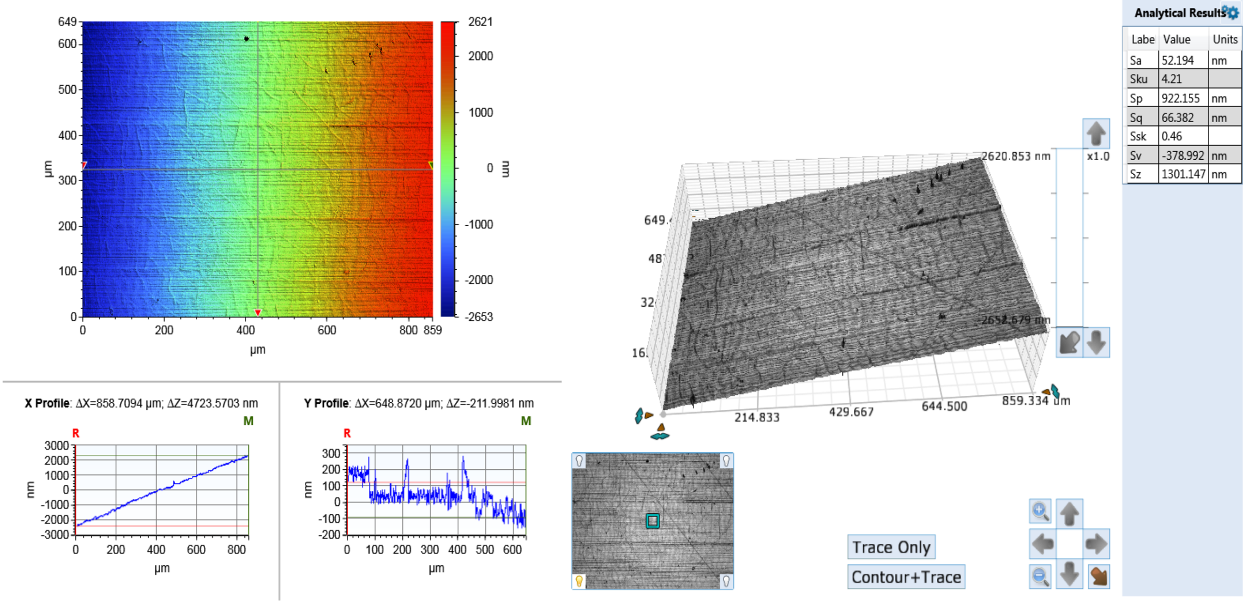

Polyphenylene sulfide (PPS), Sa = 318nm

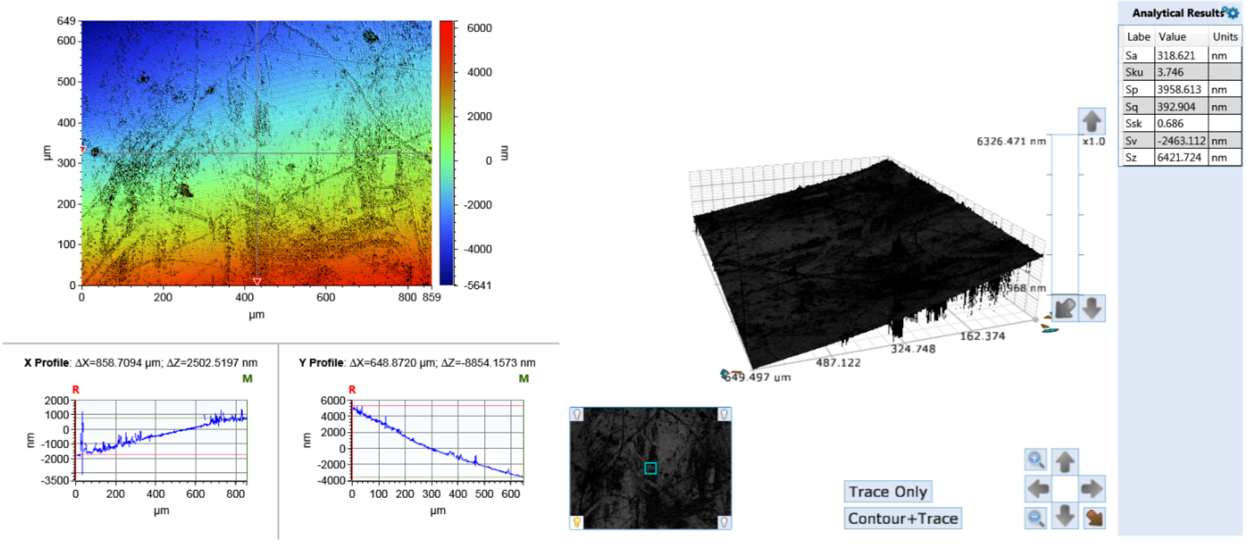

Polysulfone (PSU), Sa = 450nm

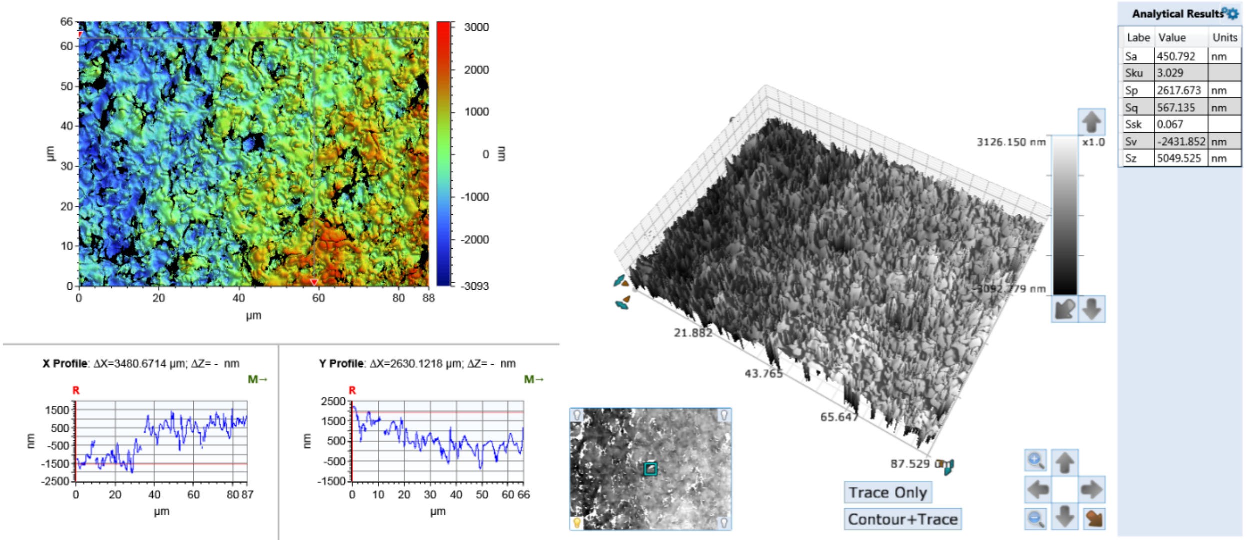

Polycarbonate (PC), Sa = 66nm

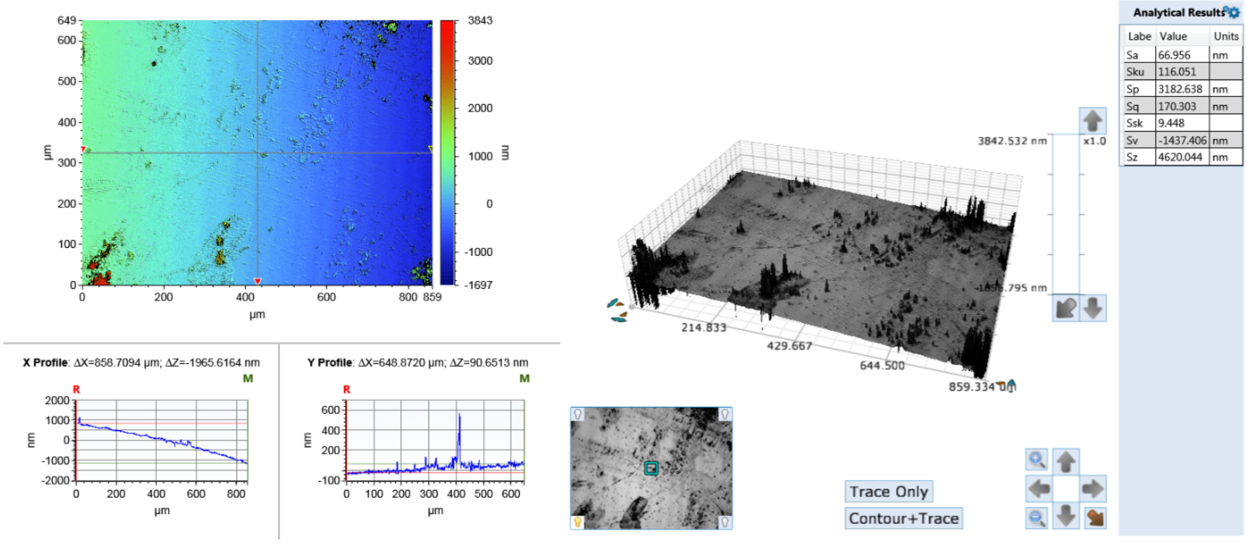

Polyethylene (PE), Sa = 243nm

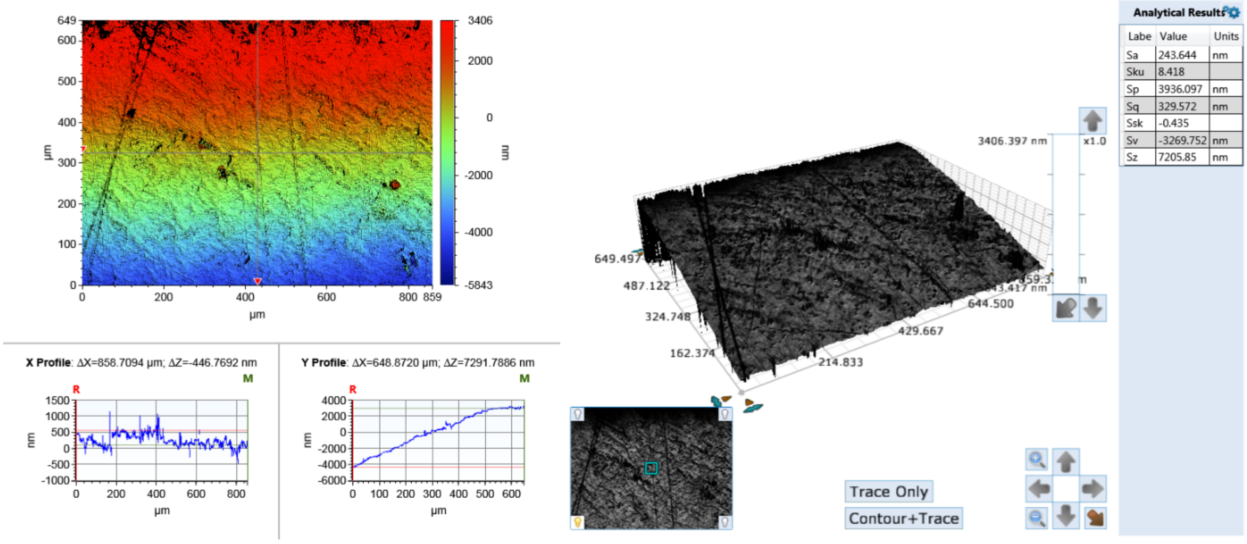

Polypropylene (PP), Sa = 89nm

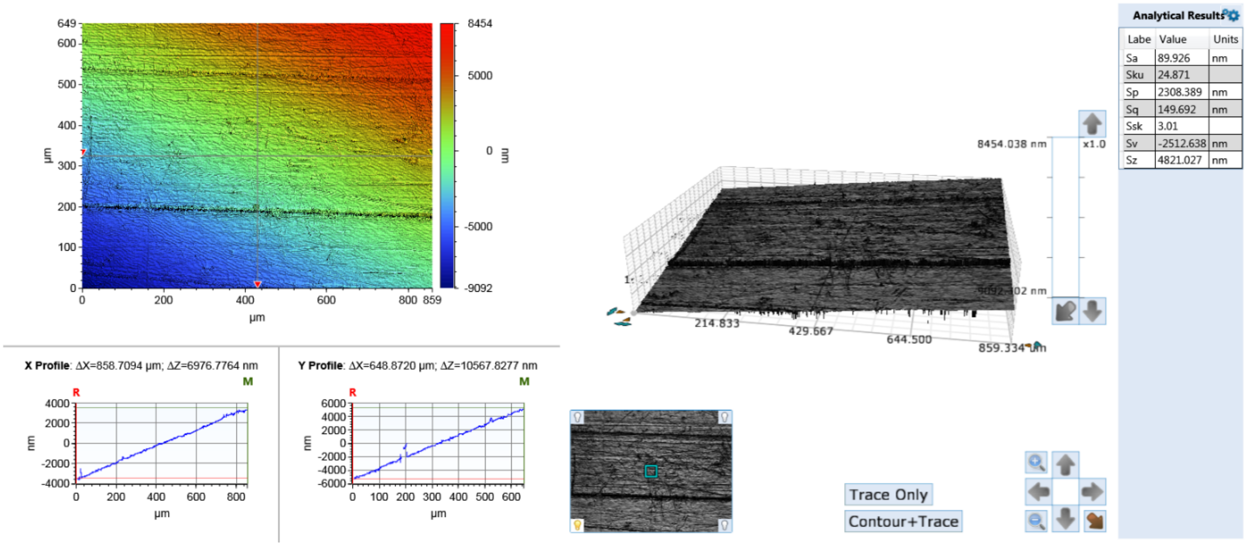

Polyethylene terephthalate (PET), Sa = 35nm

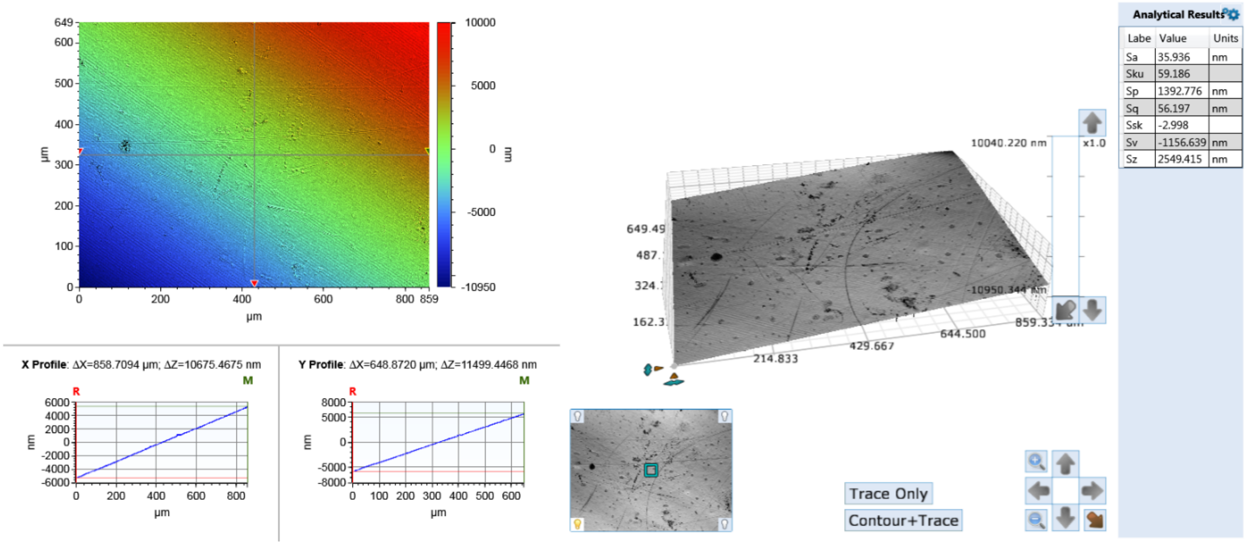

Polymethyl methacrylate (PMMA), Sa = 33nm

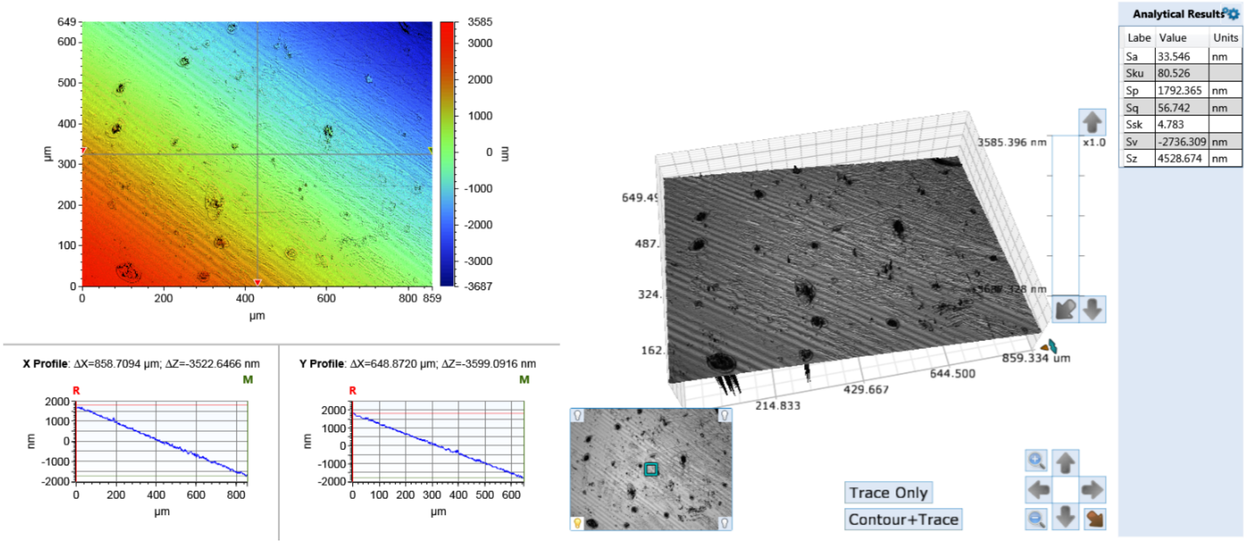

Acrylonitrile Butadiene Styrene (ABS), Sa = 96nm

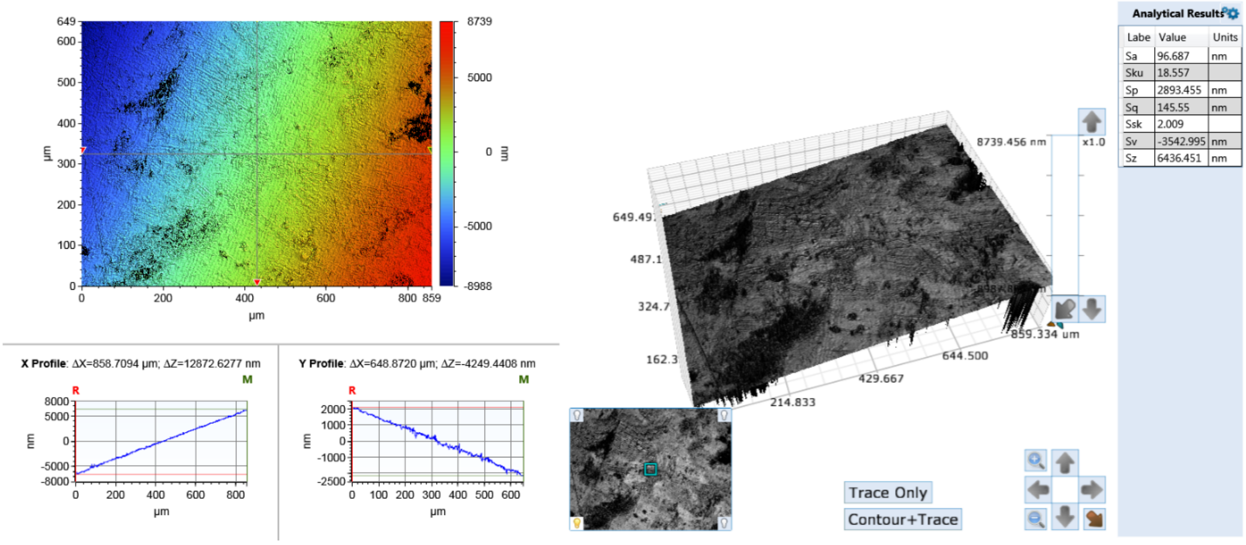

Fluorinated Ethylene Propylene (FEP), Sa = 140nm

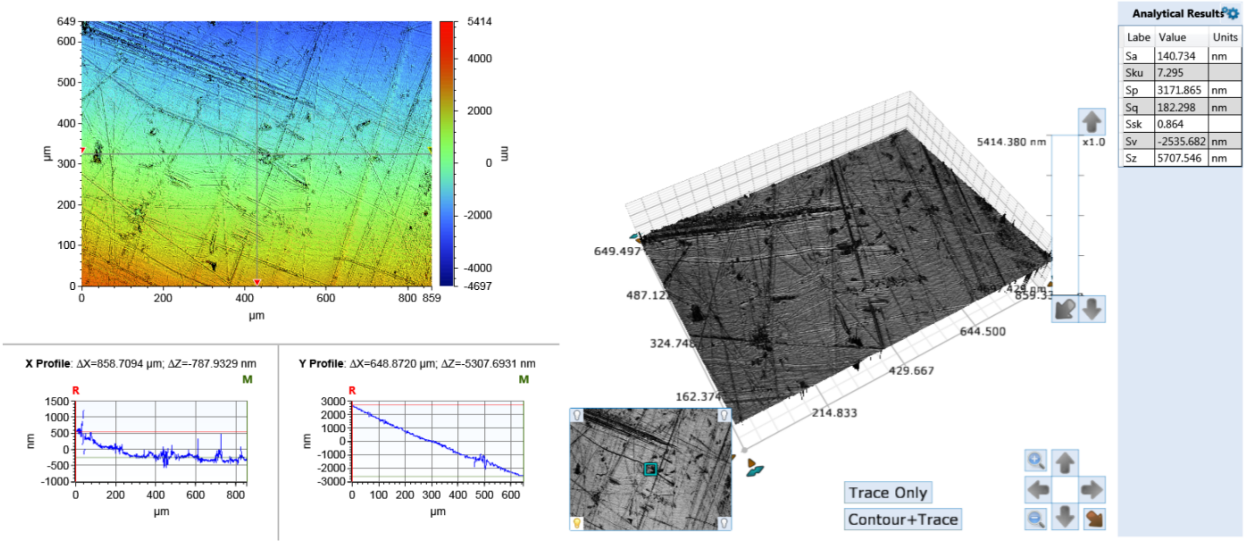

Polycaprolactum aramid 6 (Nylon6), Sa = 60nm

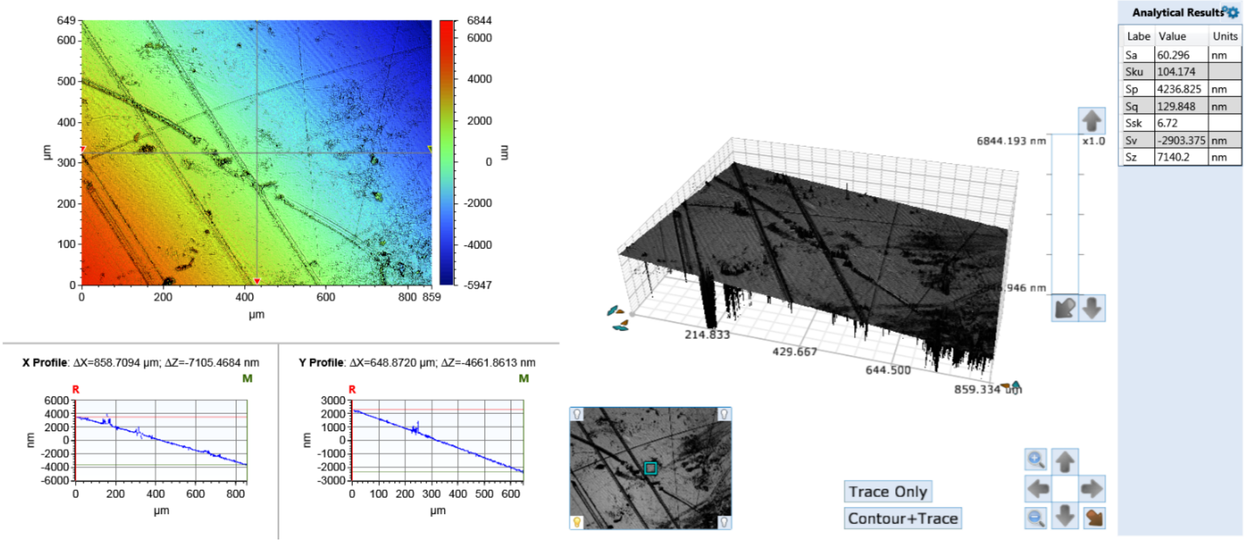

Polychlorotrifluoroethylene (PCTFE), Sa = 74nm

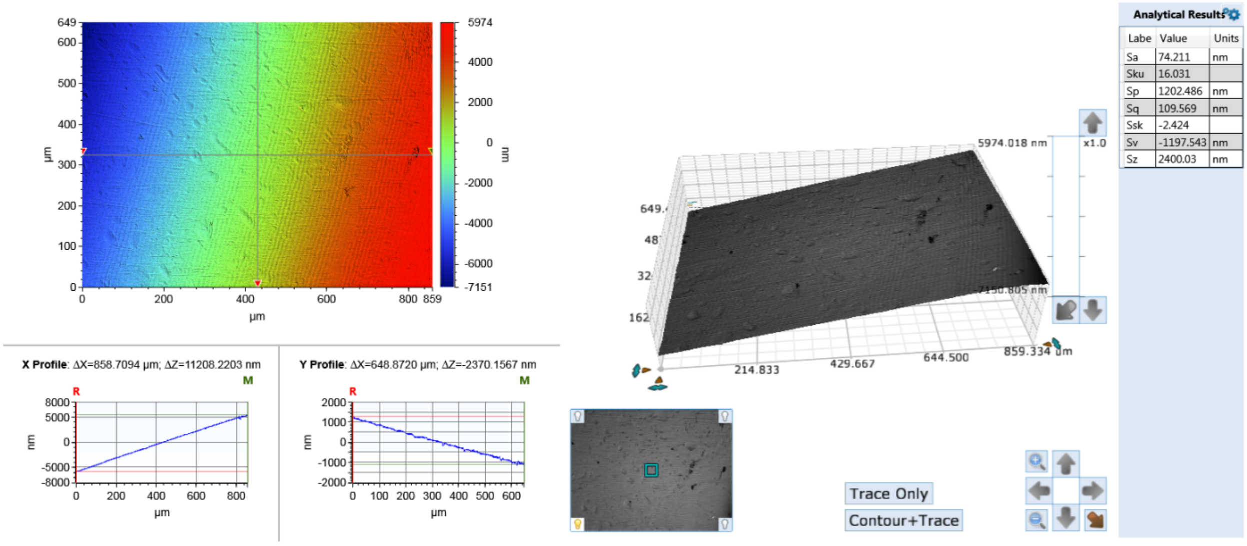

Polystyrene (PS), Sa = 33nm

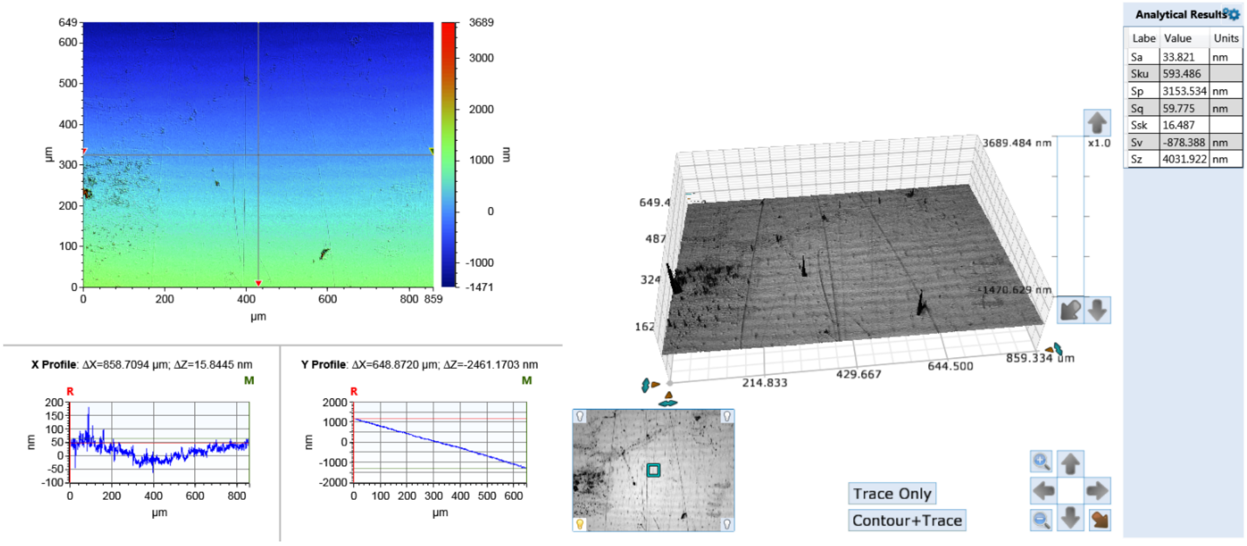

Polytetrafluoroethylene (PTFE), Sa = 462nm

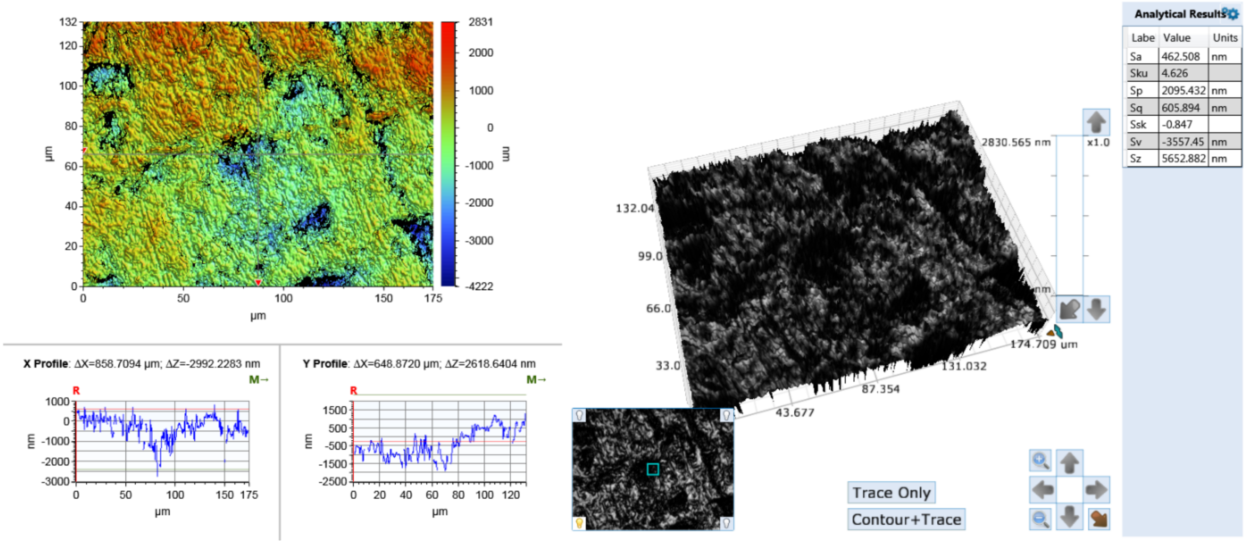

Polyvinylchloride (PVC), Sa = 43nm

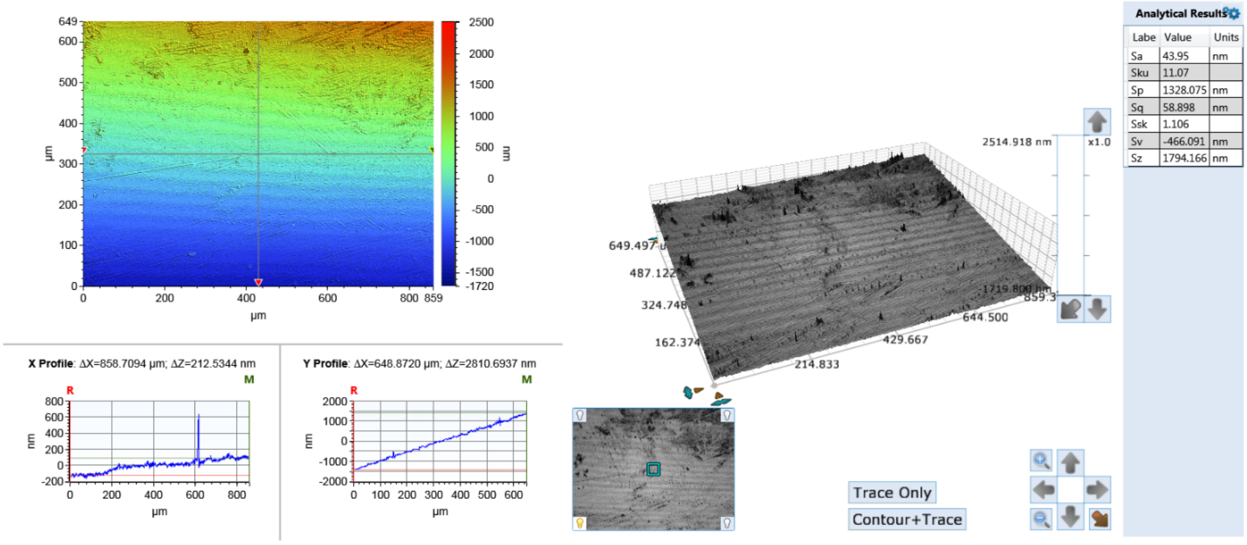

Polyvinylidene fluoride (PVDF), Sa = 62nm

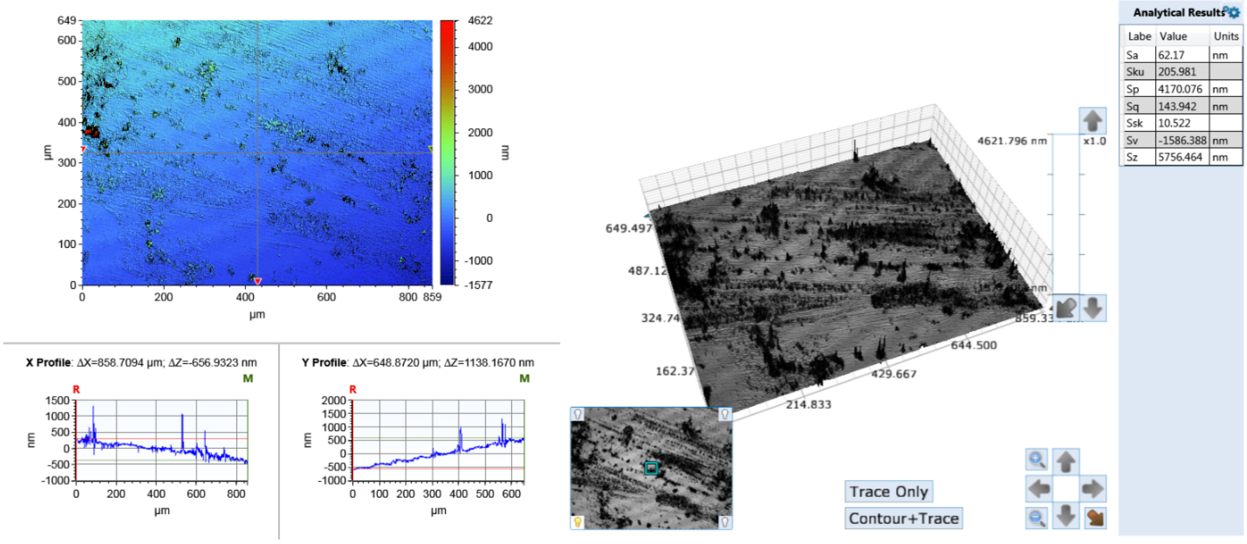

Polyether sulfone (PES), Sa = 71nm

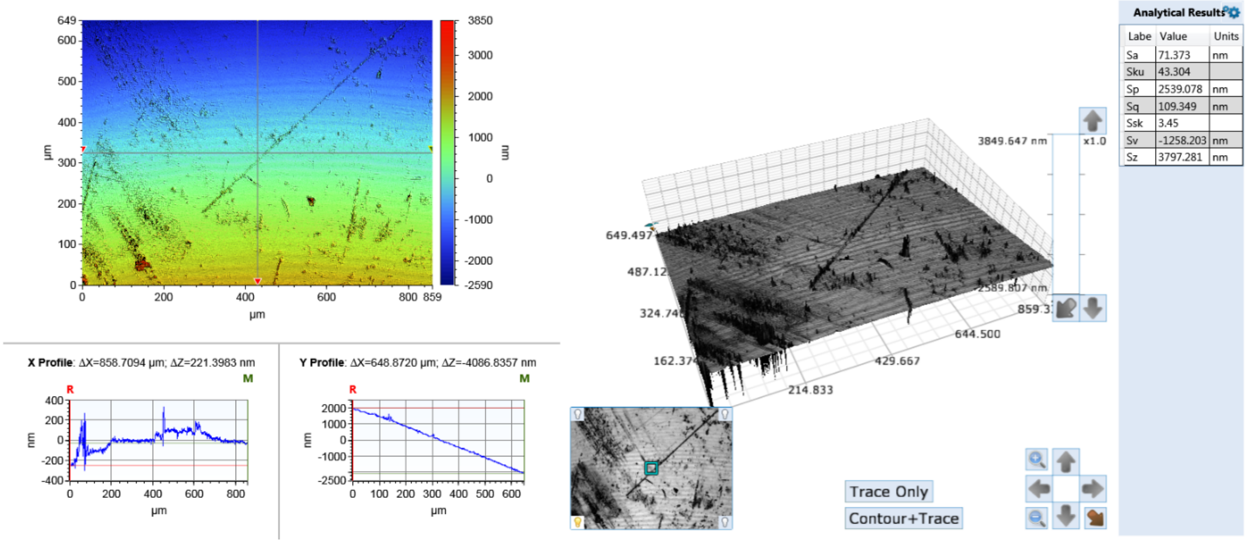

Polyphenylene oxide (PPO), Sa = 713nm

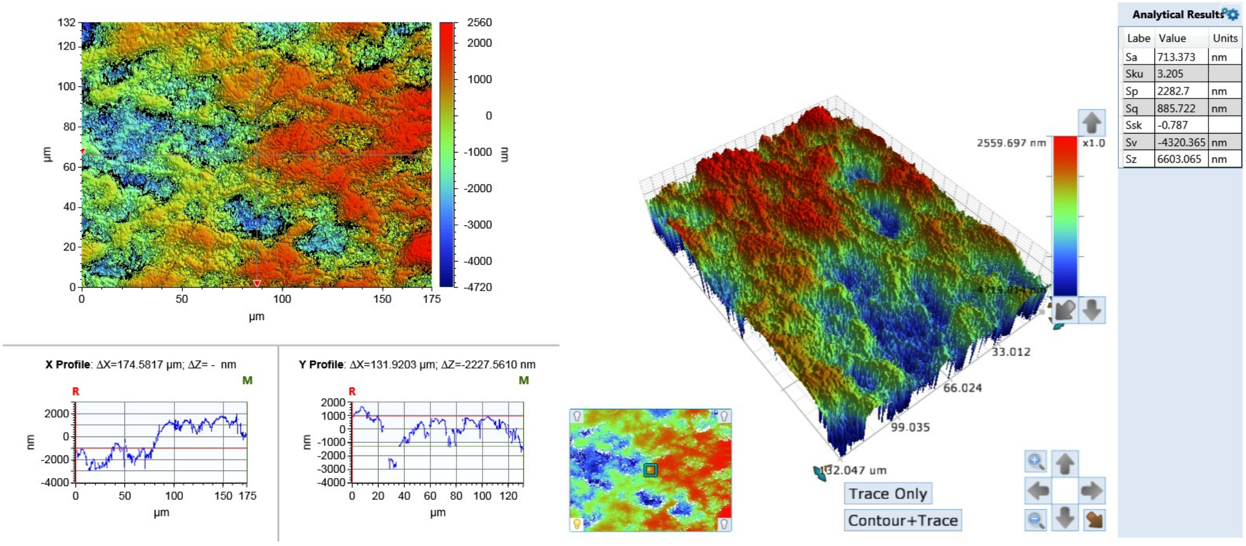

Polybutylene terephthalate (PBT), Sa = 818nm

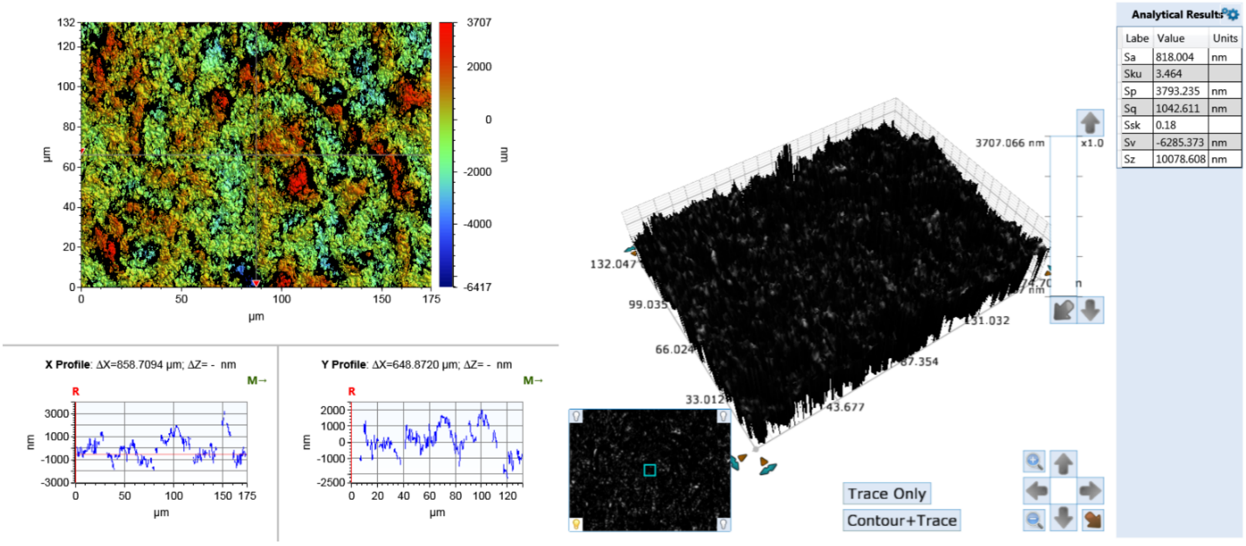

Supplementary material 3.

Example ML Code used for this work, written using Python 3.10 and PyCharm as IDE:

```
import numpy as np
from sklearn.preprocessing import MinMaxScaler
from xgboost import XGBRegressor

# Reading Data
train_data = "single_polymer_prediction/**_polymer_less_train.txt"
test_data = "single_polymer_prediction/**_polymer_only_test.txt"
print()
print(train_data)

# Training data normalization
training_data = np.loadtxt(train_data)
scaler = MinMaxScaler()
norm_data_train = scaler.fit_transform(training_data)
Features = norm_data_train[:, 1:15]  # F_1 = Polymer index
X = Features

# Scaler for wca in the training data
scaler_wca = MinMaxScaler()
reshape_wca = np.array(training_data[:, 15]).reshape(-1, 1)
norm_wca_train = scaler_wca.fit_transform(reshape_wca)
y = norm_wca_train

model_1 = XGBRegressor()  # hyperparameters exploration values goes here
model_1.fit(X, y)

# Evaluation data normalization
evaluation_data = np.loadtxt(test_data)
eval_data_reshape = evaluation_data.reshape(1, -1)  # to be used only when you have one output
```

```
norm_data_eval = scaler.transform(eval_data_reshape)  # same as above

Features_Eval = norm_data_eval[:, 3:15]
Labels_Eval = evaluation_data[15]  # to be used only when you have one output

# Making a prediction
prediction = model_1.predict(Features_Eval)

# shap testing
explainer = shap.Explainer(model_1)
shap_values = explainer(X)
shap.plots.bar(shap_values, max_display=15)
shap.plots.beeswarm(shap_values, max_display=15)

# Prediction rescaling
prediction_reshape = prediction.reshape(-1, 1)
x_pre = scaler_wca.inverse_transform(prediction_reshape)
flat_pre = list(x_pre.flat)

print()
pipeline_scores = model_1.feature_importances_
reshape_pipeline_scores = pipeline_scores.reshape(-1, 1)
print(reshape_pipeline_scores)
print()
print("Predicted WCA:", x_pre)
print()

real_reshape = Labels_Eval.reshape(-1, 1)
print()
print("Experimental WCA:", real_reshape)
print()
```

```
print('train dataset shape', X.shape)
print('evaluation dataset shape', Features_Eval.shape)
```

Additional References

(1) Przykaza, K.; Woźniak, K.; Jurak, M.; Wiącek, A. Wetting Properties of Polyetheretherketone Plasma Activated and Biocoated Surfaces. *Colloids and Interfaces* **2019**, *3*. DOI: 10.3390/colloids3010040.

(2) Al-Amshawee, S.; Yunus, M. Y. B. M.; Lynam, J. G.; Lee, W. H.; Dai, F.; Dakhil, I. H. Roughness and wettability of biofilm carriers: A systematic review. *Environmental Technology & Innovation* **2021**, *21*. DOI: 10.1016/j.eti.2020.101233.

(3) James Jose, A.; Wilson, R.; Jacob, G.; Alagar, M. Studies on thermo mechanical and surface properties of polysulfone/poly(ether imide ester) blends. *Materials Today: Proceedings* **2019**, *9*, 279-294. DOI: 10.1016/j.matpr.2019.02.159.

(4) Pickup, O. J. S.; Khazal, I.; Smith, E. J.; Whitwood, A. C.; Lynam, J. M.; Bolaky, K.; King, T. C.; Rawe, B. W.; Fey, N. Computational Discovery of Stable Transition-Metal Vinylidene Complexes. *Organometallics* **2014**, *33*, 1751-1761. DOI: 10.1021/om500114u.

(5) Kelar, J.; Shekargoftar, M.; Krumpolec, R.; Homola, T. Activation of polycarbonate (PC) surfaces by atmospheric pressure plasma in ambient air. *Polymer Testing* **2018**, *67*, 428-434. DOI: 10.1016/j.polymertesting.2018.03.027.

(6) Liu, D.; Chen, P.; Mu, J.; Yu, Q.; Lu, C. Improvement and mechanism of interfacial adhesion in PBO fiber/bismaleimide composite by oxygen plasma treatment. *Applied Surface Science* **2011**, *257*, 6935-6940. DOI: 10.1016/j.apsusc.2011.03.035.

(7) Khan, M. M.; Chapman, T.; Cochran, K.; Schuler, A. J. Attachment surface energy effects on nitrification and estrogen removal rates by biofilms for improved wastewater treatment. *Water Res* **2013**, *47*, 2190-2198. DOI: 10.1016/j.watres.2013.01.036.

(8) Peng, P.; Huang, H.; Ren, H.; Ma, H.; Lin, Y.; Geng, J.; Xu, K.; Zhang, Y.; Ding, L. Exogenous N-acyl homoserine lactones facilitate microbial adhesion of high ammonia nitrogen wastewater on biocarrier surfaces. *Sci Total Environ* **2018**, *624*, 1013-1022. DOI: 10.1016/j.scitotenv.2017.12.248.

(9) Lundberg, S., Lee, S. A Unified Approach to Interpreting Model Predictions. In 31st Conference on Neural Information Processing Systems, Long Beach, CA, USA; 2017.
